# Biological Properties and Applications of Betalains

**DOI:** 10.3390/molecules26092520

**Published:** 2021-04-26

**Authors:** Izabela Sadowska-Bartosz, Grzegorz Bartosz

**Affiliations:** 1 Laboratory of Analytical Biochemistry, Institute of Food Technology and Nutrition, College of Natural Sciences, Rzeszow University, 4 Zelwerowicza Street, 35-601 Rzeszów, Poland; 2 Department of Bioenergetics, Food Analysis and Microbiology, Institute of Food Technology and Nutrition, College of Natural Sciences, Rzeszow University, 4 Zelwerowicza Street, 35-601 Rzeszów, Poland; gbartosz@ur.edu.pl

**Keywords:** betalains, betacyanins, betaxanthins, betanidin, betanin, antioxidant, food colorants

## Abstract

Betalains are water-soluble pigments present in vacuoles of plants of the order *Caryophyllales* and in mushrooms of the genera *Amanita*, *Hygrocybe* and *Hygrophorus*. Betalamic acid is a constituent of all betalains. The type of betalamic acid substituent determines the class of betalains. The betacyanins (reddish to violet) contain a cyclo-3,4-dihydroxyphenylalanine (cyclo-DOPA) residue while the betaxanthins (yellow to orange) contain different amino acid or amine residues. The most common betacyanin is betanin (Beetroot Red), present in red beets *Beta vulgaris*, which is a glucoside of betanidin. The structure of this comprehensive review is as follows: Occurrence of Betalains; Structure of Betalains; Spectroscopic and Fluorescent Properties; Stability; Antioxidant Activity; Bioavailability, Health Benefits; Betalains as Food Colorants; Food Safety of Betalains; Other Applications of Betalains; and Environmental Role and Fate of Betalains.

## 1. Occurrence of Betalains

**Figure 1:**
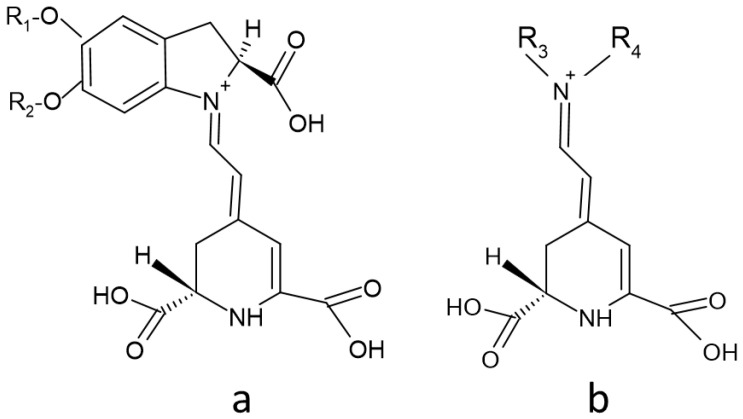
Structure of and betacyanins (**a**) and betaxanthins (S form) (**b**). R1 and R2: hydrogen or sugar moieties; R3: amine or amino acid group; R4: usually hydrogen.

Betalains are pigments of about 17 families of plants belonging to the order *Caryophyllales* [1]. Betalains can be divided into two subclasses: betacyanins (red–violet) and betaxanthins (yellow to orange) (Figure 1). Interestingly, anthocyanins and betalains, which apparently have similar/identical functions, have never been found together in the same plant so seemingly they are mutually exclusive [2]. Betalains are hydrophilic and are accumulated in the vacuoles of the cells, mainly in the epidermal and subepidermal tissues of plants which synthesize these pigments [3]. Betalains bestow color on flowers of many genera of plants, such as *Mirabilis* [4], *Glottiphylum* [5] and *Portulaca* [6]. In *Caryophyllales* anthocyanins determine the coloration in the families of *Caryophylaceae* and *Molluginaceae*.

The most known edible sources of betalains in the *Caryophyllales* are red beet roots (*Beta vulgaris* L.), grainy or leafy amaranth (*Amarathus* sp.), fruits of the cacti *Opuntia* sp., *Eulychnia* sp. and *Hylocereus* sp., among them the dragon fruits of mainly *Hylocereus polyrhizus* (Web.) Britton and Rose) and similar species [7,8,9,10,11,12,13], the colored Swiss chard (*B. vulgaris* L. ssp. *cicla*) [14], *Celosia argentea* L. [15] and *Bougainvillea* sp. [16]. Less common sources include the tubers of ulluco (*Ullucus tuberosus* Caldas) [17] and the bloodberries (berries of *Rivina humilis* L.) [18]. Some species of *Amaranthus* are consumed fresh or cooked [19,20]. Interestingly, tissues of grain amaranths such as *Amaranthus cruentus* L., *A. coudatus* L. and A. *hybridus* L. contain more betacyanins than those of *A. tricolor* L., a vegetable amaranth [21]. *Phytolacca americana* L. could also be a source of betalains, but it is not used as a food colorant since it contains toxic saponins and lectins [22]. In the *Portulacaceae* family, betalains have been reported in the common purslane, *Portulaca grandiflora* Hook. [23,24] and *Talinum triangulare* (Jacq.) Willd [25]. Surprisingly, fungi of the genera *Amanita*, such as *Amanita muscaria* (L.) Lam. (fly agaric), *Hygrocybe* and *Hygrophorus* also contain betalains [26,27,28,29,30,31]; their role in fungi is unknown. Recently a natural occcurrence of a betalain in a bacteria has been described: the bacteria *Gluconacetobacter diazotrophicus* were reported to produce dopaxanthin [32].

**Figure 2:**
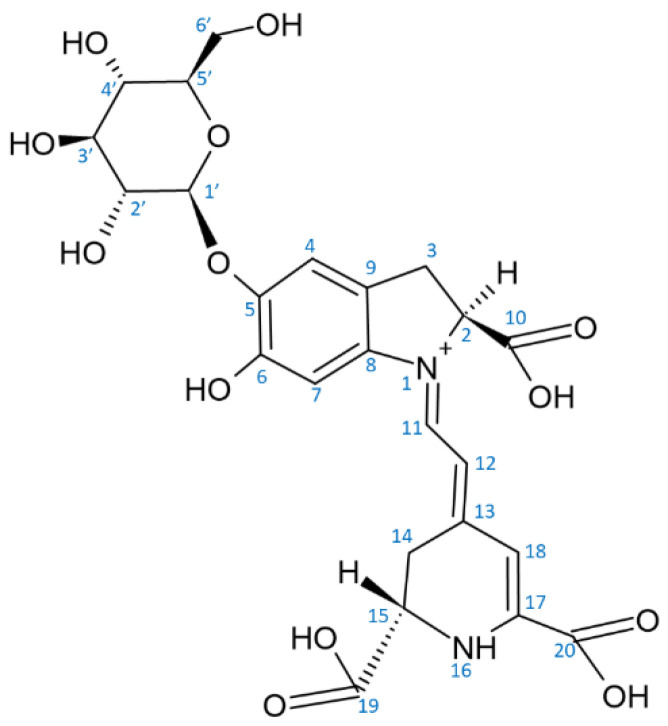
Structure of betanin (CAS 7659-95-2).

Red beet (*Beta vulgaris*), containing two major betalain pigments, the red betanin and the yellow vulgaxanthin I, has been considered for long as the unique source of betalains. Red beet is cultivated all over the world and is commonly and frequently consumed. The world production of beetroot was estimated to be about 275 million metric tons in 2018 [33]. Betacyanins constitute approximately 75–95% of beetroot pigments, the remaining 5–25% being betaxanthins [34]. The concentrations of betalains in red beetroots are 200–2100 mg/kg fresh weight. Betalain content varies significantly between cultivars [35,36]; some new varieties achieve higher contents of betalain [37,38]. Apart from the main betacyanin, betanin (betanidin 5-*O*-β-glucoside; CAS 37279-84-8, called also beetroot red (Figure 2)), red beet contains isobetanin (CAS 15121-53-6), an epimer of betanin [39]. The content of these pigments is the highest on the surface (peel) of the beetroot and steadily decreases to the interior of the root [34,40,41,42,43]. In fresh red beetroots, two betalain precursors (betalamic acid, CAS 18766-66-0 and cyclo-DOPA, CAS 18766-67-1) and eight betacyanins and their degradation products were detected: betanin > neobetanin (CAS 71199-29-6) > 2-decarboxy-neobetanin ≈ isobetanin > 2,17-bidecarboxy-betanin > isobetanin > 17-decarboxy-neobetanin > 6′-*O-*feruloyl-betanin > 6′-*O*-feruloyl-isobetanin along with two betaxanthins: vulgaxanthin I, CAS 904-62-1 > miraxanthin V (CAS 5375-64-4) (listed in order of decreasing content) [44]. The real occurrence of neobetanin has been questioned; this compound has been claimed to be an artefact of isolation, formed under acidic conditions [45]. However, not all authors share this view [40,46]. Fresh beetroot juice contains ca 1.2 g/mL betanin [47]. Betanin is also the most abundant component of the processed beetroot juice [48]. 

The beetroot, in spite of having a high betanin content, has some important drawbacks, such as: limited composition of pigments, carryover of soil microbes leading to microbial contamination, and adverse earthy flavor caused by the high content of geosmin and various pyrazines [49]. This aspect aroused interest in alternative sources of betalains, especially cacti, among them various *Opuntia* species. Such plants can be easily grown in arid/dry areas at low cost. Moreover, they can be used as a feed for ruminants even after extraction of the pigments [50].

Production of individual betalains by genetically engineered microorganisms (*Escherichia coli* and *Saccharomyces cerevisiae*) [51,52] and higher plants, such as potatoes (*Solanum tuberosum* L.), tomatoes (*Solanum lycopersicum* L.), eggplants (*Solanum melongena* L.) and petunias (*Petunia x hybrida*) has been reported [53], which points to new potential sources of these pigments. Rice (*Oryza sativa* L.) was engineered to synthesize betanin in the endosperm by introduction of three synthetic genes controlled by an endosperm-specific promoter (“Betanin Rice”) [54]. Betalain production by beet cell cultures has also been considered [55,56]. Such an approach would facilitate the control of pigment production eliminating the effects of environmental factors [57]. However, the productivity of cell cultures is much lower and costs of betalain production much higher in comparison with the beetroot, which can cheaply produce up to about 0.5 g of betanin per kg of roots [58].

## 2. Structure of Betalains

**Figure 3:**
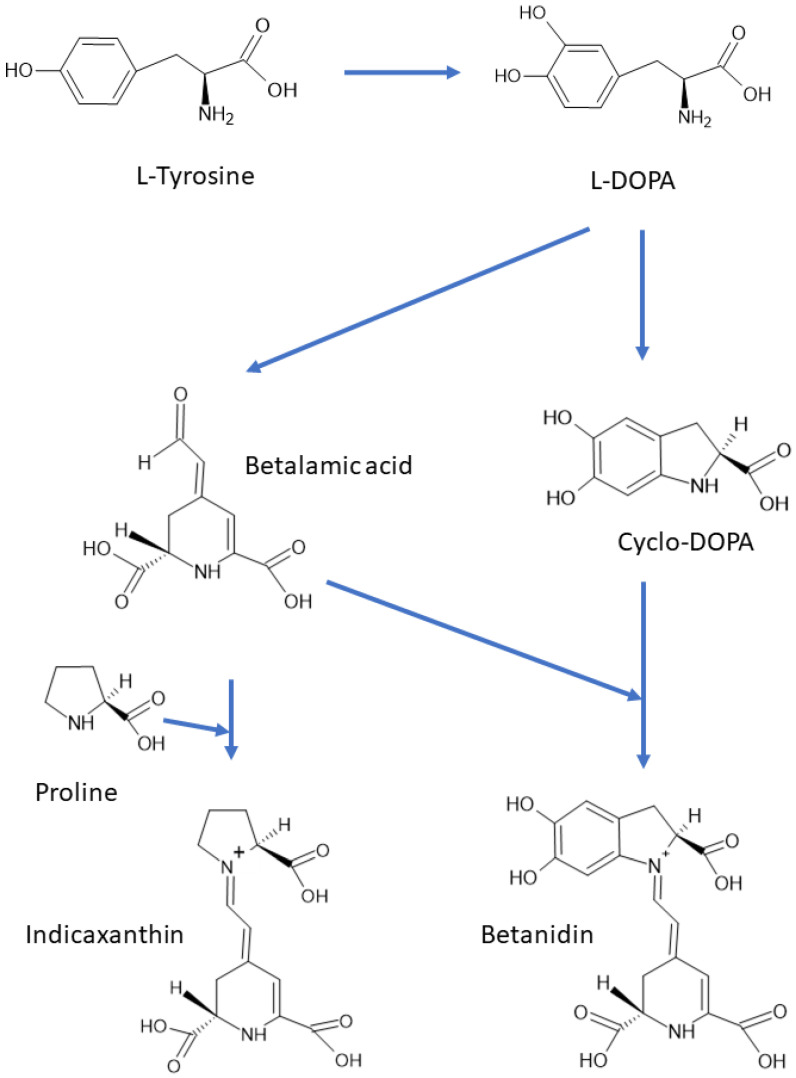
Biosynthesis of betalains. After [22], modified.

There are about 80 well characterized plant betalains [1] and 3 betalains from the fly agaric *Amanita muscaria* [50]. All betalains contain betalamic acid [4-(2-oxoethylidene)-1,2,3,4-tetrahydropyridine-2,6-dicarboxylic acid; CAS 18766-66-0] [30]. As mentioned above, the reddish to violet betacyanins are derivatives of betanidin, an iminium adduct of cyclo-dioxyphenylalanine (cyclo-DOPA) and betalamic acid, whereas the yellow to orange betaxanthins are condensation products of betalamic acid with α-amino acids or amines (Figure 1 and Figure 3). Derivatization (glycosylation and acylation) of betanidin at position 5 or 6 of leads to the formation of various betacyanins. Most of the derivatives are 5-*O*-glucosides, but 6-*O*-glucosides have also been found. No betacyanin having both positions substituted with sugar residues has been detected [34]. The 5-*O*-glucosides can be further glycosylated or esterified with hydroxycinnamic acids [30]. Derivatives of 2-decarboxy and 15-decarboxy-betanins have also been detected [30,34].

Two enzymes participate in the biosynthesis of betalamic acid: (1) a bifunctional cytochrome P450 enzyme CYP76AD1 having both monooxygenase [59] and diphenol oxidase [60] activities, and (2) 4,5-DOPA dioxygenase (DODA) or L-DOPA ring opening enzyme [61,62]. In the first step, tyrosine is hydroxylated to L-DOPA by CYP76AD1 (and/or tyrosinase), and in the second step betalamic acid is formed by the action of DODA and subsequent spontaneous cyclization. Betalamic acid condenses spontaneously with cyclo-DOPA and/or its glucosyl derivatives, amines or their derivatives, forming betacyanins or betaxanthins, respectively. Novel reactions in the biosynthetic pathway of betalains have been described, explaining the formation of specific flower pigments of various plant species [63]. Structures of most common betacyanins and betaxanthins, along with their light absorption maxima, are shown in Tables S1 and S2, respectively.

## 3. Spectroscopic and Fluorescent Properties of Betalains

Betalamic acid, due to its conjugated double bonds, is the main chromophore of all betalains [40]. The condensation of betalamic acid with cyclo-DOPA in betacyanins widens the electronic resonance onto the diphenolic aromatic ring. This extra conjugation results in a bathochromic effect, shifting the absorption maximum from about 480 nm, typical of betaxanthins, to the region of about 540 nm [1,30,64]. The type and position of betanidin substituents affect the wavelength of the absorption maximum of betacyanins.

Betacyanins have two absorption maxima, one in the UV-range (270–280 nm), conditioned by the cyclo-DOPA residue and a second one in the visible green range (535–540 nm, changing with the solvent). The position and nature of substituents determines the tint of various betacyanins. In general, betanidin glycosylation results in a hypsochromic shift (decrease in the wavelength of absorption maximum) of about 6 nm while addition of a second sugar moiety the first one does not affect the color significantly [21,40]. Attachment of glucose at the C6 position is less effective than glycosylation at C5 [65]. The absorption spectrum of betanin has a maximum at 536 nm; decarboxylation at C-2 results in a hypsochromic shift [66]. A more pronounced hypsochromic effect is observed in neobetanin (CAS 71199-29-6 = 14,15-dehydrobetanin), which is orange and not violet [67]. The same effect is found in betaxanthins: a hypsochromic shift occurs upon decarboxylation of portulacaxanthin II (CAS 135545-98-1) and dopaxanthin [68].

The effect of esterification depends on the nature of the substituent: esterification with aliphatic acyl moieties has little impact on the maximum absorption of betacyanins, while acylation with aromatic acids causes a bathochromic shift (increase in the wavelength of absorption maximum) [10,69]. This bathochromic shift was ascribed to intramolecular association, similar to co-pigmentation [70]. A third maximum in the range of 300–330 nm appears in the case of acylation with hydroxycinnamic acids [40]. In betaxanthins, attachment of different amino acids or amines may result in either hypsochromic or bathochromic shifts [30]. Conjugation with amino acids leads to higher absorption maxima in comparison with amines [9] (Tables S1 and S2).

Betalains display a broad range of colors, from yellow to red and violet. Their spectra do not change in the pH range of 3–7; however, upon lowering the pH to <3, there is a small (about 2-nm) hypsochromic shift and a hypochromic effect (decrease in absorption intensity) in the absorption band at 535–540 nm. This hypochromic effect is accompanied by a slight hyperchromic effect (increase in absorption intensity), in the range of 575–650 nm, and a color change from red to purple. Upon pH increase, changes in the wavelength of maximum absorption and a hypochromic effect are observed. The broad range of colors of the prickly pear (*Opuntia* ssp.) is due to the combination of betacyanins and betaxanthins. Betalains may complex metal ions, which can lead to either bathochromic or hypochromic effect [71,72,73].

Betalains, mainly betaxanthins, emit fluorescence. Their fluorescence spectra are characterized by excitation maximum in the range of 463–535 nm and emission maximum in the range of 508–608 nm (Table S3). The fluorescence quantum yields of some betalains are given in Table S4. Betalains fluoresce in their physiological environment, i.e., mainly in the petals. This fluorescence may play a role in the signaling between flowers and pollinators [6].

Increased fluorescence may be expected if electron density is withdrawn from a resonating system. The high fluorescence of miraxanthin I, containing methionine sulfoxide, in comparison with methionine-betaxanthin may serve as an example [74]. Another example is the higher fluorescence of vulgaxanthin II (CAS 1047-87-6), containing glutamic acid residue, in comparison with vulgaxanthin I, containing glutamine residue, caused by the electron-withdrawing effect of the carboxyl group [75]. The same effect was demonstrated for semisynthetic betalamic coumarins: substitution of the electron-donating methyl group in the coumarin moiety of the semisynthetic betalain cBeet120 by the electron-withdrawing trifluoromethyl group resulted in cBeet151, a molecule with higher fluorescence yield than that of CBeet120 [76]. The extra carboxylic group in dopaxanthin (CAS 71199-31-0) and portulacaxanthin II; CAS 135545-98-1 = tyrosine-betaxanthin) augments the fluorescence with respect to dopamine-betaxanthin (CAS 5375-64-4) and tyramine-betaxanthin (CAS 5589-85-5), respectively [74]. An opposite effect (a decrease in fluorescence) is caused by introduction of an electron-donating group (e.g., a hydroxyl group) to a betalain molecule [8]. 

Other structural considerations should also be considered. I.a., the effect of groups able to generate hydrogen bonds has been studied [77,78]. The existence of intramolecular hydrogen bonds constrains the dynamics of the molecule, favorizing certain structures, as it occurs in miraxanthin I (CAS 5296-79-7). This phenomenon decreases the radiationless energy losses and contributes to the high fluorescence quantum yield of miraxanthin I molecule (Table S4), recognized as the most fluorescent natural betaxanthin [68].

Increasing excitation wavelengths causes a decrease in the Stokes shift (the separation between excitation and emission maxima in the fluorescence spectra) [6]. In contrast to betaxanthins, which are the highly fluorescent, betacyanins show much lower fluorescence. Betanidin and the product of its glucosylation, betanin, are weakly fluorescent, with a very short excited-state lifetime (6.4 ps in water found for betanin) [79]. Stronger fluorescence is detected in betanins having carboxylic groups and lacking hydroxyl groups, as in the case of the indoline-derived betacyanin [80]. 

Fluorescent properties of betalains have found a variety of ingenious applications. 

*Detection of betalains after chromatographic separation, in food and in cells.* Betalains are components of complex mixtures in plant extracts or juices and their detection, quantification and isolation require appropriate separation methods, first of all high-performance liquid chromatography (HPLC). The UV and visible light absorption and fluorescence of betalains allow for their direct detection after separation. It is possible to detect the presence of betalains in plant and animal tissues be virtue of their fluorescence [6]. The content of betaxanthins and its changes during storage of food can be estimated on the basis of their fluorescence [81]. Changes of the betalain content in beet roots have been studied with time-resolved fluorescence [81,82]. An aqueous extract from beetroot, has been proposed as an easily available means for the imaging of cells; its additional advantage is the lack of cytotoxicity [83].

*Monitoring ingestion and digestion betalains by Caenorhabditis elegans*. The nematode *C. elegans* has been used as an animal model to study the effect of betalains administered in the diet and the digestion of betalains. Since the body of *C. elegans* is transparent, it is possible to monitor the ingested betalains by their fluorescence in the digestive tube of the nematode [84].

*Reporter activity in transgenesis.* Betalain fluorescence can be used to measure the effectiveness of transformation of model plants with genes enabling production of betalains [53]. There are attempts to generate new betalain-producing plants, which could be edible. This goal is within reach, as demonstrated by effective transfection of the model plant *Arabidopsis thaliana* (L.) Heynh. with DODA from *Amanita muscaria.* Transformed plants accumulated betaxanthins in flowers [85]. The fluorescence of betalains allows also to visualize individual transformed cells producing these pigments [86]. 

*Fluorescent detection of malaria-infected erythrocytes*. Semisynthetic betaxanthin BtC, synthesized by condensation of betalamic acid with 7-amino-4-methylcoumarin, can pass the erythrocyte membrane, bind to the intracellular parasite *Plasmodium falciparum*, and thus stain erythrocytes infected with this parasite [87]. 

*Activity assays of oxidative enzymes*. The activity of oxidases can be conveniently monitored by continuous fluorescence measurements of reactions employing betaxanthins. These compounds are substrates for such enzymes as peroxidases and tyrosinases [63]. The reaction products are weakly fluorescent, so the rate of fluorescence disappearance is a measure of the rate of betaxanthin oxidation [88]. 

*Monitoring tyrosine hydroxylase-dependent biosyntheses in the yeast.* Betaxanthin fluorescence enables monitoring of a range of biosynthetic activities provided they can be linked to a reaction producing betalamic acid. In some plants, alkaloids derived from L-DOPA, which is transformed through a series of reactions into (*S*)-reticuline, are the starting point for the biosynthesis of a wide variety of substances, such as codeine, morphine, and other opioid drugs [89]. Yeast transfected with genes coding for several relevant biosynthetic plant enzymes can produce benzylisoquinoline alkaloids from L-DOPA. The yeast *S. cerevisiae,* although able to synthesize L-tyrosine, cannot accumulate L-DOPA. Transfected yeasts are able to produce L-tyrosine and then appropriate drugs from added L-DOPA [90]. It is possible to detect L-DOPA production by introducing DODA to the system. DODA converts L-DOPA into betalamic acid, which condenses spontaneously with amines or amino acids forming fluorescent betaxanthins [89,90]. The fluorescence of betaxanthins can be used to identify L-DOPA-producing strains using L-tyrosine as a substrate. The same idea has been exploited to identify strains of *S. cerevisiae* producing L-tyrosine with high yields [91].

*Labeling of proteins*. Betalamic acid reacts with any free amine group available. Free amine groups present in proteins can thus be labeled with betalamic acid to form yellow protein-betaxanthins, of spectral and fluorescence properties analogous to low-molecular weight bethaxanthins. It is also possible to substitute betalamic acid as the labeling reagent with red beet root juice subjected to a simple degradation procedure involving in situ alkalization and neutralization. Such a reagent stains proteins separated by electrophoresis. Thus, one can cheaply stain proteins in electrophoretic gels with easily processed red beet root juice [92].

*Output Signal in Biosensors*. By virtue of their fluorescence, betalains can serve as biosensors. An example of such application of betalains is the use of whole-cell *Cupriavidus metallidurans* sensors for the analysis of environmental copper [93]. Such an approach is not limited to metals; this new field of application of betaxanthins is quite broad since betaxanthin fluorescence can be used as the output signal of virtually any sensing device, provided it involves an inducible promoter which can activate the expression of DODA in the presence of L-DOPA.

## 4. Betalain Stability

Degradation of betalains may proceed via different mechanisms. Betalain stability is affected by various factors, both intrinsic and extrinsic, which should be taken into account when applying these compounds in the food industry [94] (Table 1).

*Temperature stability*. The most important factors affecting the stability of betalain during storage and food processing are temperature and pH. At pH 7, betanin was stable for at least 20 days when stored at 4 °C and for more than 275 days in the frozen state (at −30 °C) [47]. Betalains are sensitive to heat and degrade at temperatures above 50 °C; this feature is a significant drawback in their use for the coloring of food [95,96,97]. Degradation of betacyanins at high temperatures leads to formation of yellow products, including neobetacyanins, betalamic acid, and newly formed betaxanthins [94]. Boiling of betalain-containing plant material decreases the content of both betacyanins and betaxanthins. The red color of a betanin solution gradually fades and turns into yellowish-brown upon heating at 100 °C [98,99]. Boiling of betanin-containing material evokes an increase in the percentage contribution of dehydrogenated and decarboxylated betanin derivatives, accompanied by elimination of the glycoside [33]. Thermal degradation of betanin solutions as well as red beet and purple pitaya juices follows first-order reaction kinetics [73,95,99,100]. Red beet boiling for 60, 120 and 180 s decreased the amount of betacyanins by 6%, 22% and 51%, respectively, and that of betaxanthins by 18%, 23% and 33%, respectively [101]. Another study revealed that 60-min boiling of red beet roots decreased betalain content by 51% [44]. Other authors reported a decrease of red beet content of betacyanin by 24%, 62% and 81% and of betaxanthins content by 13% 60% and 73%, after 30-min treatment at 105, 115 and 125 °C, respectively [102]. 

Interestingly, antioxidant activity of the beet increased rather than decreased during heat treatment [101] suggesting that betalain degradation products may show enhanced antioxidant properties. When betaxanthin content was heat-degraded to 60%, total phenolic content and antioxidant activity increased slightly because of decomposition of betalain to phenolic compounds showing higher antioxidant activity than their native precursors [103,104].

**Figure 4:**
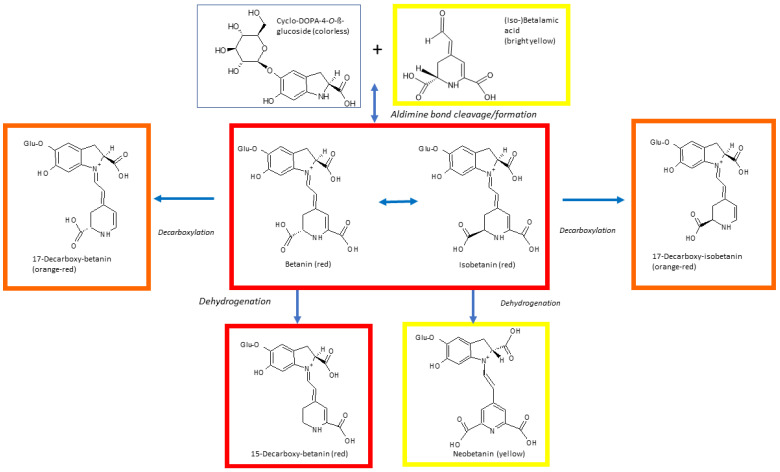
Some degradation pathways of betanin. After [111], modified.

Dehydrogenation of betanin occurring during heating leads to formation of neobetanin, responsible for the change of color to yellow. Cleavage may also generate the yellow betalamic acid and the colorless cyclo-DOPA-5-*O*-glycoside. The color of betanin is preserved upon C15-isomerisation or decarboxylation but C17-decarboxylation results in a hypsochromic shift of the absorption maximum from 538 to 505 nm, which is seen as an appearance of an orange-red color [105] (Figure 4). 2-Decarboxy and 15-decarboxy-betanins are also formed. Comparison of thermal degradation of betanin in three model solvent systems (water/glycerol, water/ethylene glycol and water/ ethanol), in the temperature range of 60–86 °C revealed that the stability of betanin was the lowest in the water/ethanol system. As ethanol has a high electron density on the oxygen atom, this result supports the hypothesis that the initial step of the thermal degradation of betanin is the nucleophilic attack on the aldimine bond [106]. Other studies confirmed fast degradation of betacyanins in ethanolic solutions and occurrence of single and double decarboxylation. Interestingly, monodecarboxylation products were different in ethanolic solutions and in aqueous solutions of betacyanins, suggesting different decarboxylation mechanisms, depending on the solvent. In red beetroot juice and purple pitaya extract, decarboxylated neo-derivatives were also detected among other betacyanin degradation products induced by heating [107,108]. All mono- and bi-decarboxylated betacyanins formed upon heating red beet and purple pitaya preparations have been identified [109]. Studies of the degradation products of betanin, hylocerenin (3-hydroxy-3-methylglutarylbetanin: CAS 403517-96-4) and phyllocactin (malonylbetanin; CAS 15167-85-8) formed during heating of purple pitaya juice showed that the hydrolytic cleavage was the main degradation mechanism of betanin, while decarboxylation and dehydrogenation were predominant in hylocerenin. Phyllocactin degradation was the most complex, and included decarboxylation of the malonic acid moiety, generation of betanin by demalonylation and betanin degradation. Upon prolonged heating, an additional double bond at C2–C3 appeared in the degradation products [110].

Esterification of betacyanins with aliphatic acids was reported to enhance their stability. Lower susceptibilities of phyllocactin and hylocerenin towards hydrolytic cleavage compared to betanin suggests protection of the aldimine bond by aliphatic acid moieties. Phyllocactin and hylocerenin, possessing dicarboxylic acid residues, are also decarboxylated upon heating [110]. The decarboxylation does not affect the positions of the light absorption maxima. As a result, hylocerenin solutions show a higher apparent chromatic stability towards thermal degradation than betanin solutions, which, however, is due to a higher stability of the heat-induced degradation products of hylocerenin in comparison with the parent compound. Phyllocactin solutions are less stable [112] due to presence of the malonic acid residue, which is susceptible to cleavage of the carboxyl group at the β-position and to deacylation. The thermal stability of betacyanins may also be enhanced by esterification with aromatic acids. This effect is caused by intramolecular stacking, since the U-shape folding of the molecule prevents the aldimine bond from hydrolytic attack. The position of the aromatic acid influences the stability; 6-*O-*esterification is more stabilizing than 5-*O*-esterification [70,113]. Substitution with aliphatic acids also increases the thermal stability of betacyanins [97,114]. However, most of the reported data based on spectrophotometric measurements may not reflect the real stability of the parent compounds if their degradation products do not have lower absorbance. Stability assessment on the basis of HPLC analysis did not confirm some conclusions drawn from spectrophotometric measurements, e.g., betanin was found by HPLC to be more stable than the acylated structures [112]. 

Several papers pointed to a higher stability of betacyanins in comparison with betaxanthins at room temperature [115,116] and upon heating [94,105,116]. For example, the half-life of thermally treated betanin was 11 times longer than that of vulgaxanthin I [116]. Comparison of thermal stability of *Amaranthus* betacyanins and red radish *Raphanus raphanistrum* subsp. *sativus* (L.) Domin anthocyanin in model food systems showed that betacyanins, having brighter red color than the red radish anthocyanin, showed color stability at 14 and 25 °C similar to the anthocyanin, but betacyanin color was less stable than that of red radish anthocyanin at 37 °C [117].

*pH stability*. As anthocyanins are unstable at pH values above 3 [40], and betalains are relatively stable in a pH range of 3–7 [64], betacyanins are the natural pigments of choice if foods of low acidity are to be stained red to purple. The pH range of 5–6 is optimal for maximum betanin stability [71,72,98,118,119]. Alkaline conditions induce aldimine bond hydrolysis, while acidification causes recondensation of betalamic acid with the amine group of the substituent residue [119,120]. C15 isomerization [121] and dehydrogenation [122] were observed under acidic conditions. Betanin degradation rate under fluorescent light was found to be three-fold higher at pH 3 than at pH 5 [73]. Although activation energy for betacyanin degradation decreases with increasing pH in the range of 3–5, the rate constant of degradation decreases as well, so pigment application to most foods undergoing ordinary thermal treatments is not impaired, e.g., losses of betacyanin in pitaya juice acidified to pH 4 was less than 10% during 5-min pasteurization at 80 °C [118]. The dependence of betalain stability on pH is affected by several factors. High temperatures shift the pH optimum for betacyanin stability toward 6 [96] so while under anaerobic conditions the pH optimum for the stability of betanin being 5.5–5.8, it is shifted to lower pH (4.0–5.0) in the presence of oxygen [72,123]. Both alkaline and acid conditions induce betanidin decomposition into 5,6-dihydroxyindole-2-carboxylic acid and methylpyridine-2,6-dicarboxylic acid [124]. Betanin solutions are much less stable at pH 2 than at pH 3 [73]. Low pH induces C15 isomerization of betanin and betanidin into isobetanin and isobetanidin (CAS 4934-32-1), respectively [122,125], and formation of the yellow neobetanin [122,126]. However, the spectral changes (small hypsochromic and hypochromic shifts in betanin absorbance peak at pH values below 3.0 accompanied by a slight hyperchromic effect at 570–640 nm) [64] cannot be fully explained by any of the degradation mechanisms reported so far. 

Betaxanthins have maximal stability in the pH range of 4–7 [127], with the pH optimum of 5.5. Vulgaxanthin I is less stable [128] and more susceptible to oxidation [129,130] than betanin at acidic pH values. Under alkaline conditions indicaxanthin (CAS 2181-75-1) generates protocatechuic acid [131], which prevents regeneration of the betaxanthin. 

*Metals, oxygen and antioxidants*. Some metal cations, such as Fe^2+^, Fe^3+^, Cu^2+^, Al^3+^, Sn^2+^ and Cr^3+^ impair betalain stability accelerating their degradation [132,133,134,135]. Chelating agents, such as citric acid and ethylenediaminetetraacetic acid (EDTA) stabilize betanin against metal-catalyzed degradation [128,132,134,136,137], by binding metal ions, but perhaps also by a partial neutralization of the positively charged amino nitrogen in the electrophilic center upon association with these pigments [134,135,137]. The presence of metal complexing compounds in plant extracts or juices provides protection against this effect of metal ions as reported for the beet juice, which is less susceptible to metals than a pure betanin solution [135].

Betalains react with molecular oxygen [138]. The kinetics of aerobic degradation of betanin deviates from that of the first-order reaction in absence of oxygen, which is attributed to the reversibility of this reaction, not observed in the presence of oxygen. The stability of betalains is improved by a nitrogen atmosphere [94,138,139,140]. Storage of betalain solutions under low oxygen levels can decrease the degradation of the pigment [72,73]. 

Apparently, the aerobic degradation of betacyanins is mediated by reactive oxygen species (ROS). Glycosylation was found to increase 17 times the half-life value of betanidin and isobetanidin exposed to degradation by ROS. This effect was explained by a lower redox potential of betanidin as compared to betanin [119]. Oxidative stability of betacyanins can be improved by further glycosylation: betanin and amaranthin (betanidin 5-*O*-[2’-*O*-(β-glucuronic acid)]-β-glucoside; CAS 15167-84-7) exhibited identical thermal stability in the presence of oxygen while under anaerobic conditions amaranthin was less stable than betanin [123].

As expected, stability of betalain is improved by antioxidants [136,138,141]. Some food antioxidants, especially ascorbic acid and isoascorbic acid, were reported to enhance betalain stability [117,137,139,142,143]. However, a prooxidant effect of ascorbic acid a concentration of 1000 mg/kg was also observed [134]. This discrepancy can possibly be explained by the betalain bleaching caused by hydrogen peroxide generated during oxidation of ascorbic acid [128]. Results of studies concerning the replacement of ascorbic with isoascorbic acid are divergent. Some authors found that isoascorbic acid stabilizes betanin better than ascorbic acid [138,144] while others reported a better effect of ascorbic on the retention of the pigment [137]. Moreover, as ascorbic acid has a negative effect on the stability of anthocyanins [145,146], betalains rather than anthocyanins can be recommended for coloring foods with high ascorbic acid contents [94,143]. Phenolic and sulfur-containing antioxidants, acting by termination of free radical reactions were ineffective in betanin protection, which suggests that betanin oxidation of betanin does not involve a chain free radical mechanism [138]. 

*Light and UV radiation*. Absorption of UV radiation or visible light excites π electrons of the pigment chromophore to a more energetic state (π*), thus increasing reactivity of a molecule [64]. Light exposure impairs betalain stability; this effect increases with increasing light intensity in the range of 2200–4400 lux [21,147]. The light-induced degradation of betalain is oxygen dependent, and under anaerobic conditions the effects of light exposure are negligible [123,147]. Comparison of stability of three red pigments to UVB radiation showed that betanin was the least stable, less stable than carminic acid and a synthetic dye, Red 40 [148].

*Concentration/water activity*. The concentration of betalains is also a key factor determining their stability during food processing. Betalain degrade easily when extracted and stored in the form of a solution; their stability increases with increasing pigment concentration [149,150]. For concentrated matrices, water activity is a more appropriate parameter than betalain concentration. Betalain stability decreases exponentially with increasing water activity, most probably because it determines the susceptibility of the pigments to aldimine bond cleavage [94,129]. Another factor may be the reduced mobility of reactants and limited oxygen solubility in matrices of low water activity [34]. Low water activity improves betalain stability, the water activity below 0.63 being the most effective [150]. Betalain stability is also affected by water activity during processing [109]. Hence, spray drying or convective drying is recommended to avoid colorant degradation [151,152,153]. 

*Enzymatic degradation*. Red beets contain endogenous enzymes such as β-glucosidases, peroxidases and polyphenoloxidases, which may cause degradation of betalain and color loss if not properly inactivated by blanching [154,155,156]. Enzymatic degradation of both betacyanins and betaxanthins has a pH optimum around 3.4 [157]. The products of enzymatic degradation are similar to those of thermal, acid or alkaline degradation [156]. Betalains are degraded by peroxidases; the presence of catalase, decomposing hydrogen peroxide, the co-substrate for peroxidases, suppresses betalain oxidation [158]. The hydrolysis of betanin glycosides and release of aglycones causes a bathochromic shift of 4–6 nm. However, the aglycones are more labile and prone to oxidation, so the loss of red color and subsequent browning follow betanin hydrolysis [40]. Acylation of betalains prevents pigment cleavage by endogenous or exogenous β-glucosidase [159,160], allowing for better color retention upon enzymatic treatment during food processing.

*Regeneration*. When extracts containing the primary degradation products of betanin are kept for some time at a temperature below 10 °C and pH around 5.0, betanin may be spontaneously regenerated [72,98]. Betanin regeneration occurs via a partial resynthesis of betanin from the hydrolysis products and condensation of the amine group of cyclo-DOPA-5-*O*-glycoside with the aldehyde group of betalamic acid; upon mixing these two compounds in solution, betanin is quickly formed [98].

*Fermentation.* Fermentation of red beets decreases the betalain content [44]. Lactic fermentation was also reported to promote isomerization and dehydrogenation of betanin. Still, aglycons can be formed from betanin by fermentation, and the betanidin/betanin ratios depend thus on the endogenous β-glucosidase activity of a cultivar [161].

*Encapsulation*. Encapsulation is an efficient way of stabilization, easy administration and improvement of bioavailability of betalains [162,163]. Various methods of encapsulation have been employed. Encapsulation with polysaccharides (pectin or guar gum) as the wall material enhances betalain stability [164]. *Amaranthus* betacyanins were encapsulated using maltodextrin (MDE) as the carrier and starch as the coating agent; such encapsulation enhanced pigment stability and reduced loss of color during a storage period of four months [152]. Encapsulating *Opuntia lasiacantha* Pfeiffer betanin extract with MDE reduced pigment loss by up to 14% during a 6-month storage [165]. Encapsulation of miraxanthin V (CAS 5375-64-4) and betanidin with chitosan and maltodextrin limited pigment loss and allowed for retention of antioxidant activity after 6-month storage [166]. Encapsulating purified indicaxanthin with MDE preserved the color intensity and prevented pigment degradation during storage and 20 °C for more than six months [167]. Indicaxanthin-containing extract of the cactus pear (*Opuntia* sp.) encapsulated with MED or inulin was more stable than encapsulated betacyanin at 60 °C [168].

## 5. Antioxidant Activity

Both structural components of betalains (a phenolic and a cyclic amine) are good electron donors, so betalains have antioxidant properties [169]. The antioxidant and antiradical activities of betanin have been analyzed and explained in terms of the electron donor capacity, bond dissociation energy and the ionization potential of the molecule [170]. Owing to resonance, the secondary amine group is conjugated with the hydroxyl group participating in the keto−enol tautomeric equilibrium of the formyl group. The withdrawal of an electron from the phenolic oxygen of betacyanins is relatively easy, and the betacyanin radicals formed are stabilized by the delocalization of the unpaired electron through the aromatic ring. The connection of the betalain characteristic electron resonance system to the aromatic ring increases the Trolox equivalent antioxidant capacity (TEAC) of betalains by 0.4 mol/mol betalain [15].

Betalamic acid is the main reactive group of betalains [169]. The TEAC value of betalamic acid estimated with ABTS^•^ at pH 7.0 is 2.7 ± 0.2 (i.e., one molecule of betalamic acid can react with 2.7 times more free radicals than a Trolox molecule), a value equal to that of resveratrol (TEAC = 2.7). At lower pH values the ABTS^•^-scavenging activity of betalamic acid is lower but still their TEAC is >1. This pH dependence of reactivity of betalamic acid is due to the protonation equilibrium, with a pK_a_ value of 6.8 [80]. 

In betacyanins, glycosylation usually reduces the antioxidant activity, while acylation increases this activity [40,171]. Antioxidant activity of betalains is increased if a hydroxyl group is located at the C-5 position of the aglycone [172,173]. Betaxanthins can donate only an electron from the conjugated π-orbitals, which is more difficult due to the positive charge present on the nitrogen atom [173]. Thus, betaxanthins are weaker antioxidants than betacyanins. However, betaxanthins have higher numbers of hydroxyl and imino residues, which can increase their ability to scavenge free radicals. The mean TEAC of 15 betalains without aromatic resonance, charge or hydroxy groups was 2.4 ± 0.1 mol Trolox equivalents/mol. The mean TEAC of betaxanthins with charge and no aromatic resonance was 1.8 ± 0.1. Betalains with an aromatic ring in resonance with the electronic system supported by the nitrogen had a mean TEAC value of 2.8 ± 0.4. Betalains in which such resonance is combined with a second cycle fused in an indoline manner (betacyanins), had a mean TEAC increased to about 4. The presence of phenolic hydroxyl groups enhanced significantly the antiradical activity in comparison with the dehydroxylated analogs; this effect was smaller when one of the two hydroxyl groups was glucosylated [47,172].

Antioxidant properties of betalains include their ability to react with ROS; these reactions are predominantly the reactions of reduction. Cyclic voltammetry showed three anodic waves for betanin and two anodic waves for indicaxanthin. The peak potentials characterizing the reducing power of these compounds were: 404, 616, and 998 mV for betanin and 611 and 895 mV for indicaxanthin. The shape of the voltammetric curves indicated that both betalains are irreversibly oxidized and their products cannot accept additional electrons [174].

Betanin was shown to dose-dependently scavenge ABTS, DPPH, galvinoxyl, superoxide, peroxyl and (of course) hydroxyl free radicals [175] and was found to have the highest antioxidant activity from among 17 betalains studied [84].

The radical-scavenging activity of betalains depends on pH due to the pH-dependent protonation/deprotonation. In the ABTS^●^ scavenging assay, both betacyanins and betaxanthins show greater antiradical activity at neutral and basic pH than at acidic pH, which indicates that the deprotonated species are mainly responsible for the antiradical properties of betalains. The stoichiometry of the ABTS^●^ scavenging by betanin increases with increasing pH, from a value of about 0.2 mol ABTS^●^/mol at pH 2, to about 7 mol ABTS^●^/mol at pH 7 and about 9 mol ABTS^●^/mol at pH 9 [171,172]. This dependence can be ascribed to sequential ionization of appropriate carboxyl groups in the betanin molecule. At pH < 2 the cationic form of a betalain dominates; at pH = 2 a zwitterion form of net charge zero prevails, in the pH range of 2.0–3.5 a form of net charge –1 with deprotonated C2-COOH and C15-COOH groups is the most abundant, at 3.5 < pH < 7.0 dianion (net charge = 2) with deprotonated C2-COOH, C15-COOH and C17-COOH groups predominates, and at 7.0 < pH < 9.5 most molecules exist as trianions (net charge –3) with deprotonated all carboxyl groups and protonated phenolic C6-OH group [176]. Another comparative study considering 15 natural and synthetic compounds confirmed this pH dependence of the free radical scavenging activity of betalains [143]. Other authors found values of 20 and 1.76 ABTS^●^/mol for betanin and indicaxanthin, respectively, at pH 7 [174]. In the FRAP test (which is performed at pH 3.6), each betalamic acid molecule reduced two Fe(III) ions to Fe(II) [173].

Comparison of DPPH^●^ scavenging activities of betalains isolated from plants of the *Amaranthaceae* family showed the following sequence of IC_50_ values (concentrations scavenging 50% of DPPH^●^ radicals in 60 µM DPPH^●^ solution): (iso)gomphrein I, 3.35 µM < acylated gomphreins, 4.11 µM < betain, 4.88 µM < isobetain, 4.89 µM < celosianins (acylated amaranthine), 7.13 µM < iresinins (acylated amaranthine), 8.08 µM < isoamaranthine, 8.35 µM < amaranthine, 8.37 µM. In this system the IC_50_ value for ascorbic acid was 13.93 µM), indicating better antioxidant properties of betalains in comparison with ascorbic acid [171].

Betanin, phyllocactin and betanidin scavenged peroxyl radicals generated by AAPH with the stoichiometry of 3.31 ± 0.14, 2.83 ± 0.01 and 10.70 ± 0.01 mol Trolox equivalents/mol, respectively. All these compounds scavenged nitric oxide (betanidin > phyllocactin > betanin) [177]. Betanin inhibited the peroxynitrite-dependent nitration of tyrosine and was more effective than ascorbate (IC_50_ values of 19.2 μM and 79.6 μM, respectively). Betanin (0.05–1.0 mM) concentration-dependently inhibited DNA strand cleavage induced by peroxynitrite, scavenged nitrogen dioxide and was a lipid peroxyl radical-scavenger [178]. Thus, betalains can protect cells not only from oxidative stress but also from nitrosative stress [179]. Betanin and indicaxanthin are good electron donors for myeloperoxidase (MPO) compounds I and II. They interfere with the catalytic cycle of the enzyme and can enhance its chlorination activity at verylow concentrations and pH 7.0. This activity is, however, accompanied by the ability of betalains to scavenge HOCl. At pH 5.0, when the release of HOCl by MPO is maximal, both betalains act as scavengers already at low micromolar concentrations. Betanin react with hypochlorite and are chlorinated at C-19 [180]. The apparent second order rate constant for the reaction of betanin with hypochlorite is (1.8 ± 0.2) × 10^4^ M^−1^s^−1^ at pH 7.0 and 25 °C [181]. Betanin quenches singlet oxygen with a rate constant of (1.20 ± 0.15) × 10^8^ M^−1^. The quenching proceeds by a chemical mechanism; the main product of betanin oxidation is 2-decarboxy-2,3-dehydrobetanin [182]. 

Peroxidation of linoleic acid induced by cytochrome c was inhibited by of betanin and betanidin at very low concentrations. The IC_50_ values for the inhibition of peroxidation of linoleic acid by betanin, betanidin, catechin, and R-tocopherol were 0.4, 0.8, 1.2, and 5 µM, respectively, demonstrating that the betalains studied are better inhibitors of lipid peroxidation than tocopherol and catechin. Betaxanthins also inhibited lipid peroxidation induced by cytochrome c with IC_50_ values of about 1.0 µM. In the process of metmyoglobin-catalyzed peroxidation of linoleic acid, the heme is oxidized and subject to destruction, which leads to decrease in absorbance at the Soret band. Betanin prevented both lipid peroxidation and heme destruction. Simultaneously, pigment absorbance at 542 nm decreased, evidencing destruction of the double-bond structure of betanin [169]. Oxidation of low-density lipoprotein (LDL) by H_2_O_2_ + metmyoglobin was also inhibited by betanin, with IC_50_ < 2.5 µM, lower than that of catechin. The inhibitory effect of betanin on microsomal lipid peroxidation was dependent on the oxidant used. When peroxidation was induced by ascorbic acid + FeCl_3,_ low concentrations of betanin (5 and 12.5 µM) acted pro-oxidatively and only 25 µM betanin inhibited lipid oxidation. However, when lipid peroxidation was caused by H_2_O_2_ + metmyoglobin, betanin concentrations <5 µM almost totally inhibited microsomal peroxidation. Betanidin and betanin were efficient inhibitors of soybean lipoxygenase-induced peroxidation of linoleate (IC_50_ values of 0.3, 0.6, and 1.2 µM, respectively) [169]. Betanin was also effective in preventing lipid peroxidation in meat (ground pork loin) stored at 4 °C for 9 days; 2% betanin was as effective as 0.1% butylated hydroxyanizole (BHA) or butylated hydroxytoluene (BHT) [47].

Binding of betalains to LDL both in vitro [183] and in vivo [184,185] has been demonstrated. Bound betalains directly protected LDL from oxidation. When volunteers consumed 500 g of *Opuntia* cactus pear fruit pulp, containing 28 and 16 mg indicaxanthin and betanin, respectively, indicaxanthin was incorporated into LDL at a level of 98 pmol/mg LDL protein after 3 h. LDL isolated from their blood after this time showed a marked elongation of the lag time preceding the onset of oxidation induced by Cu^2+^ with respect to the homologous LDL isolated before eating the fruit (58 min vs. 40 min). No changes in the LDL-bound vitamin E and β-carotene were noted in this experiment so the effect seemingly could be attributed to indicaxanthin [185].

Betanin interacts with Cu^2+^ ions, forming a complex with a 1:2 ratio; such binding may prevent the prooxidant action of Cu^2+^. No such interaction was observed for indicaxanthin [174].

Human erythrocytes showed increased resistance to oxidative hemolysis induced by cumene hydroperoxide after incubation in vitro with indicaxanthin and betanin, under conditions leading to incorporation of 0.45–1.1 nmol betalains per mL of packed cells.

Indicaxanthin and betalain accumulated also in erythrocytes of volunteers who consumed 500 g of fresh cactus pear fruit pulp. Peak concentrations of indicaxanthin and betalain in erythrocytes (1.03 and 0.03 µM, respectively) were achieved 3 h after consumption; at this time, resistance of erythrocytes to oxidative hemolysis was considerably increased. Again, consumption of the cactus pulp did not increase the content of vitamins C and E and glutathione in erythrocytes; on this premise the authors concluded that the increase in erythrocyte resistance to oxidative hemolysis was due to betalain absorption [186]. 

In Huh7 cells betanin protected DNA against H_2_O_2_-induced damage, as estimated by the comet assay, transactivated the nuclear factor (erythroid-derived 2)-like transcription factor; Nrf2) at a concentration of 15 µM as well as heme oxygenase 1, a down-stream target of Nrf2 (at 25 µM), transactivated the antioxidant enzyme paraoxonase 1 (at 15 µM) and increased the level of glutathione (at 15 µM) [175,187]. Betanin may activate the mitogen-activated protein kinase (MAPK) pathway [187]. Betanin (2–500 µM) reduced the intensity of the respiratory burst of human polymorphonuclear leukocytes (PMNs). This compound also decreased the production of ROS and DNA damage in PMA-stimulated PMNs although it increased DNA damage in non-stimulated cells and activated caspase 3 in both stimulated and non-stimulated PMNs [188]. 

There are many studies devoted to the antioxidant activity of plant homogenates or juices. Comparison of antioxidant capacities of juices of 18 beetroot samples/cultivars, measured by ABTS^•^ scavenging, showed a considerable variation in the antioxidant capacity (10.2–21.7 μmol Trolox equivalents/mL) and content of betacyanins (0.57–1.63 mg/mL) and betaxanthins (0.31–0.95 mg/mL). A significant correlation was demonstrated between the antioxidant capacity and the content of betacyanins and a less tangible relationship between the antioxidant capacity and the content of betaxanthins, indicating that mainly betanin contributes to the antioxidant capacity of red beets [189]. It should be taken into account, however, that betalains make small contribution to the total antioxidant capacity (TAC) of fruit juices. Comparison of the content of phenolics, betaxanthins, betacyanins, and ascorbic acid with the TEAC and Oxygen Radical Absorbing Capacity (ORAC) values in the fruit pulp and juice of various *Opuntia* spp. clones showed that great differences in the contents of betaxanthins and betacyanins in various clones were accompanied by only moderate changes in TEAC and ORAC values of the pulp and juice; the contribution of betalains to TAC was the more difficult to assess that changes in their contents ran in parallel to changes in the contents of total phenolics and ascorbic acid [190] (Table S5). Moreover, it should be kept in mind that the antioxidant capacity of a given plant material can be quite variable. Comparison of 927 samples of major vegetables employing one antioxidant assay revealed 3- to 10-fold differences in the ORAC values for the same vegetable depending on the location and the season of harvest [191].

## 6. Bioavailability

A drawback of betalains, curtailing their physiological potential, is their low bioavailability [192]. After a single oral administration of 2 μmol indicaxanthin/kg body weight to rats, maximal plasma concentration of indicaxanthin of 0.22 μM was found, and the terminal elimination half-life (T_1/2_) was 1.15 h. Bioavailability of indicaxanthin in rat urine was 21% [193]. Betacyanins underwent intensive degradation when fermented red beet juice was administered intragastrically to rats and were absorbed from the stomach. Nineteen betacyanins, among them 8 native compounds and 11 metabolites, were identified in physiological fluids of the animals. Peak concentration of betacyanins was observed in the portal vein 15 s and 30 s after introduction of the extract (0.86 and 12.35 µM for 5 mg and 20 mg betacyanins, respectively). Excretion of betacyanins in urine was also the highest after 15 and 30 s (0.14 and 3.34 µmol/h after introduction of 5 and 20 mg betacyanins, respectively) [194]. In another rat study, administered betanin was metabolized mainly in the stomach wall (74%), colon (60%) and small intestine (35%). Only a small fraction of betanin was metabolized in the liver, and about 2.7% of unmetabolized betanin was excreted in urine and feces [195].

Volunteers consuming a polyphenol-free diet, who received 300 mL of red beet juice containing 120 mg of betanin, excreted 0.5–0.9% of the betanin in urine. The pigment was detected in urine from 2 to 4 h after ingestion up to 12 h; no products of betalain metabolism were found. Most of betanin remained in the gastrointestinal tract (GI) where it may exert its antioxidant action [169]. Another group was not able to detect betanin in blood plasma at any time point after ingestion of either 250 mL of beetroot juice, containing about 194 mg betanin or 300 g of whole beetroot, with about 66 mg betanin [196]. Other authors confirmed betalain recovery (about 2%) in the rat urine after ingestion of a garambullo *Myrtillocactus geometrizans* (Mart.) fruit [197]. Yet another study pointed to a higher bioavailability of betalains. When volunteers consumed 500 g of cactus pear fruit pulp, containing 28 mg indicaxanthin and 16 mg betanin, both compounds were found in their blood plasma after 60 min, attained peak concentrations after 3 h (0.20 µM for betanin) and disappeared from plasma at 12 h. Both compounds were disposed according to the first-order kinetics, the half-lives of their elimination being 2.36 h for indicaxanthin and 0.94 h for betanin. During 12 h, 76% of indicaxanthin and 3.7% of the consumed betanin were excreted in urine [185]. The difference in the betalain pharmacokinetics between rat and human points to the necessity of interspecies extrapolations. 

Differences in the bioavailability of betanin from various dietary sources as estimated by urinary excretion may be significant: betanin of the cactus pear *Opuntia* sp. fruit showed a considerably greater bioavailability (3.7%) [185] than that of red beet: 2.7% [193], 0.7% [167] or 0.28% [175]. These results point to the effect of food matrix on the bioaccessibility of betalains.

Results of in vitro studies suggest that betanin degradation takes place mainly in the intestine. A decrease in the betanin content by about 54% was noted after simulated digestion with the gastric fluid; subsequent simulated intestinal digestion decreased the betanin content by additional 11% [47]. Digestibility studies employing dissected pieces of small intestine demonstrated that 26% to 60% of betalains, especially betacyanins, were metabolized, which points to a possible reason for their poor bioavailability [195,197]. Other in vitro simulations of oral, gastric, and small intestinal phases of betanin degradation indicated an about 50% pigment loss. Seemingly, indicaxanthin is almost not degraded in the GI tract. In vitro simulations of oral, gastric, and small intestinal digestion showed that only minor quantities of indicaxanthin were lost through all these digestive steps [198].

Case studies provided independent proofs of metabolic transformation of betalains [199,200]. A case of purple colon was reported in an autopsy of a patient, who consumed large quantities of red beet as an auxiliary treatment and ascribed to a beetroot pigment [199]. The beetroot pigment was identified analytically as the cause of colon discoloration in a person who died a few hours after consuming beetroot [200]. 

Studies using Caco-2 cells as a model of trans-epithelial transport of betanin confirmed that betanin can be absorbed in the intestine without any metabolic transformations and that betanin absorption can be selectively hindered by the multidrug resistance-associated protein 2 (MRP2)-mediated active efflux. Results of these studies provided further evidence that the red beet betanin is less readily absorbed compared than the cactus pear betanin, apparently due to the matrix effect [201]. Absorption of indicaxanthin by red blood cells has been reported [186].

It was reported that the bioavailability of betalains may be enhanced by complex formation with such antioxidant metals as selenium, which stabilizes betalains. Ascorbic acid facilitates the formation of the betalain-Se complex. Such complexes are probably absorbed via a Se absorption route, preferring organic Se complexes over free inorganic Se [1].

## 7. Health Benefits

Dietary betalains may play an important role in maintaining human health because of their many beneficial activities. There are numerous data on the health effects of betalains. However, in majority of cases the experiments consist in administration of plant extracts or other preparations, containing not only betalains but also a plethora of other bioactive compounds. Although it is highly probable that the observed effects are indeed due to betalains, it is usually by no means certain. The proper experimental design to prove the action of betalains should consist in either administration of purified compounds or control administration of extracts devoid of betalains. Having in mind this reservation, several intriguing observations made on crude plant extracts are still worth mentioning, at least as suggestions for further, more precise studies.

*Antimicrobial activity*. Betalains have been demonstrated to have antiviral, antibacterial, antifungal and antiprotozoal activity. Betalain-containing extracts of *Opuntia matudae* Scheinvar were found to inhibit the growth of *Escherichia coli* [202]. Beetroot (*Beta vulgaris* L.) pomace inhibited the multiplication of *Salmonella typhimurium, Staphylococcus aureus* and *Bacillus cereus* [203]. In another study, beetroot pomace was demonstrated to also inhibit the growth of Gram-negative bacteria (*E. coli, Pseudomonas aeruginosa, Citrobacter freundii, Citrobacter youngae, E. cloacae, Salmonella typhimurium*), *S. typhimurium* and *C. freundii* [204]. Betalains-rich extracts from red pitahaya (*Hylocereus polyrhizus*) inhibited Gram-positive bacteria (*B. cereus, S. aureus*, *E. faecalis*, *Listeria monocytogenes*) at 7.8 µg/mL, Gram-negative bacteria (*E. coli, Proteus mirabilis, Proteus vulgaris, P. aeruginosa, Salmonella typhi* Ty2, *Yersinia enterocolitica, Klebsiella pneumonia, Enterobacter cloacae, E. aerogenes*) at 15.6–62.5 µg/mL, yeasts (*Candida albicans, Rhizoctonia solani*) at 125–250 µg/mL and molds (*Fusarium oxysporum, Cladosporium herbarum, Botrytis cinerea, Aspergillus flavus*) at 500 µg/mL [205]. The antimicrobial activity of betalains may involve their effects on the structure, permeability and other functions of the cellular membranes of the microorganisms, which can eventually result in cell death [204]. Gram-positive bacteria generally show higher sensitivity to red beetroot than Gram-negative ones [206]. Replication of dengue virus type 2 in Vero cells was inhibited by betacyanins isolated from red spinach (*Amaranthus dubius* Mart. ex Thell.) and red pitahaya (*Hylocereus polyrhizus*), although the IC_50_ values were rather high (126.70 and 106.80 μg/mL, respectively) [207].

Betalain-rich extracts of *Amaranthus spinosus* L. and *Boerhaavia erecta* L. exhibited dose-dependent antimalarial activity in an in vivo mouse model assay. Probable mechanisms of the antiprotozoal action of betalains include inhibition of the intracellular choline transport of parasites and intracellular chelation of cations required for parasite growth (Ca^2+^, Fe^2+^ and Mg^2+^). *A. spinosus* and *B. erecta* have been used in the traditional medicine against malaria [208].

*Anticancer activity*. Anticancer properties of the beetroot were first postulated by the Hungarian physician A. Ferenczi who used it for the treatment of cancer in early 1950s. Betalains have been patented in the United States as a component of anticancer drugs (Patent number US2002178399) [33]. 

Numerous experiments demonstrated cytotoxicity of betalains and betalain-containing plant extracts towards cancer cell lines. Although no toxicity has been associated with the consumption of betalains (at concentrations of up to 100 μM) and these compounds are generally nontoxic to proliferating non-cancerous cell cultures under conditions of 24-h or longer exposure [209,210,211], growth of some cell cultures may be inhibited by betalains [187]. Indicaxanthin was found to be cytotoxic to endothelial cells [209] and Caco-2 cells [212] at concentrations above 10 μM; the betaxanthin fraction of extracts from the berries of *Rivina humilis* inhibited the growth of hepatic carcinoma (HepG2) cells (IC_50_ of 12 μM) [211]. The cytotoxic effect of betalains depends on the cell type and duration of exposure. Cytotoxicity of betanin at concentrations above 25 μM against hepatoma and adenoma cells has been reported [175,187]. Furthermore, toxicity of betanin from *B. vulgaris* (IC_50_ of 360 μM [213] or 200 μmol/L [187] to HepG2 cell cultures has been observed during prolonged exposure. Betalain isolated from *Opuntia ficus-indica* Mill. induced apoptosis of human chronic myeloid leukemia cells (line K562), with an IC_50_ for inhibition of cell proliferation of 40 µM [214]. A limited cytotoxicity of *Beta vulgaris* extracts against human breast cancer and prostate cancer cell lines was reported [215]. 

Extracts of *Opuntia ficus-indica* inhibited growth and induced apoptosis in several ovarian, cervical, and bladder cancer cells lines but also in immortalized ovarian and cervical epithelial cells [216]. Extracts from the fruit of the cactus *Hylocereus polyrhizus* concentration-dependently hampered growth of melanoma cells. The effect of peel extracts was higher compared to flesh extracts, which could be ascribed to the higher content of betalains and flavonoids in the peel. Pure betanin also strongly inhibited the growth of melanoma cells [217]. In another study, a beetroot extract rich in betanin/isobetanin was found to be cytotoxic to human breast cancer cells, with IC_50_ values of 25 µM for B16F10 and MCF-7 cells, 35 µM for MDA-MB-231 and human endothelial cells, and no significant effects on human colorectal cells and fibroblasts after 48-h treatment. Treatment with the extract activated both intrinsic and extrinsic pathways of apoptosis in breast cancer cells [211]. Other authors found betanin to be cytotoxic against various cancer cell lines (MCF-7, HCT-116, AGS and NCI-H460) with IC_50_ values in the range of 142–164 μg/mL [218]. The cytotoxic action of betanin (IC_50_ of 40 μM) on human chronic myeloid leukemia cell line (K562) involved such cellular events such as release of cytochrome c from mitochondria into the cytosol, cleavage of poly (ADP-ribose) polymerase (PARP), downregulation of Bcl-2 and diminution of mitochondrial membrane potential [214]. In human lung cancer cell lines betanin induced activation of initiator caspase-9, effector caspases-3 and 7, and cleavage of PARP, a target protein of caspase-3 [219]. 

Indicaxanthin was found to be both pro- or anti-apoptotic depending on the concentration, apoptosis-inducing agent and type of treated cells. In macrophage cultures, indicaxanthin (2.5 μM) exerted an anti-apoptotic action, including inhibition of overexpression and the basal activity of NADPH oxidase-4 (NOX-4), repression of NF-κB and maintaining cellular redox balance, Ca^2+^ homeostasis and mitochondrial membrane potential [220]. However, in colorectal carcinoma (Caco-2) cells, the same compound (115 μM) stimulated apoptosis reactivating the promoter of an onco-suppressor gene (p16INK4a) and increased synthesis of its protein product controlling the cell cycle [212].

Betalain-rich aqueous extracts of *Opuntia ficus-indica* fruits inhibited ovarian tumor growth in nude mice. Extracts of fruits of this cactus were as effective in the reduction of tumor size as a model chemopreventive agent *N*-(4-hydroxyphenyl)retinamide [216].

Betalain-rich extracts were also found to inhibit carcinogenesis. Oral administration of *Beta vulgaris* extract in drinking water to mice decreased significantly the number of papillomas in the skin after topical tumor induction. The same extract, administered orally, reduced the number of mice with adenomas (by 60%) and the number of tumors in affected animals (by 30%) after induction of lung tumors [215]. Orally given *Beta vulgaris* extracts inhibited also the formation of skin tumors induced by UV radiation or chemical treatment and reduced splenomegaly. Administration of a red beet extract containing 0.0025% betanin in the drinking water decreased the incidence of liver tumors by 60% and reduced the splenomegaly [221].

Administration of betanin (2.5 mg/100 mL water) for 25 weeks reduced (by 40%) the incidence pulmonary tumors initiated by of 4-nitroquinoline-l-oxide (4-NQO) and promoted by glycerol. Skin tumors induced by 7,12-dimethylbenz(a)anthracene (DMBA) and promoted by UVB, as well as splenomegaly were also considerably inhibited [222]. This dose of betanin inhibited significantly other tumors as well, including hepatic tumors induced by *N*-nitrosodiethylamine and promoted by phenobarbital (the 2-stage hepatocarcinogenesis model). On the basis of these findings, the possible use of betanin for combating malignancy has been suggested [221]. The molecular mechanism of anticancer activity of betanin includes inhibition of angiogenesis and induction of apoptosis by enhanced expression of caspase-3 [219]. In the interesting tumor model of *Caenorhabditis elegans* strain JK1466, tumor growth was inhibited by betanin, indicaxanthin, tryptophan-betaxanthin and phenylethylamine-betaxanthin. Interestingly, the sequence of anti-tumor effectiveness of the betalains studied did not correspond to the sequence of their antioxidant effectiveness in vitro and in vivo [223]. The mechanism of the anticancer action of betalains is still not clear, but it has been demonstrated that betalains affect the expression of some genes related to cell growth and apoptosis [216]. 

*Anti-lipidemic effects*. Rats fed betalain-rich beet crisps had significantly lowered blood serum glucose level, atherogenic index and isovaleric acid level, cecal weight and body weight [224]. Administration of red pitahaya *Hylocereus polyrhizus* fruit (300 mg/kg body weight) to hypercholesterolemic rats lowered serum lipids and total cholesterol, apparently due to enhancement of bile acids excretion [225]. In dyslipidemic rat models, suppression of the synthesis of short-chain fatty acids and prevention of increase of total serum cholesterol level by betalains was also reported [224]. Betalain-rich extracts of *Amaranthus tricolor* L. (200 and 400 mg/kg body weight) to diabetic rats decreased the levels of blood cholesterol, triglycerides and LDL, and increased the level of high-density lipoproteins (HDL) [226]. It was reported that solid betalain formulation prepared from red beet effectively maintained the serum lipid profile, increased the ratio of HDL cholesterol to LDL cholesterol and lowered the level of oxidized LDL [227]. 

Volunteers who consumed 250 g of broiled edible pulp of prickly pear *Opuntia robusta* JC Wendl. ex Pfeiffer daily for 4 months were found to have lowered total cholesterol and LDL-cholesterol levels and decreased levels of plasma and urinary of 8-epi-PGF2a, a marker of oxidative stress, the effect being stronger in females than in males [228]. Betalains from red beet juice and chips mitigated neutrophil oxidative metabolism in vivo in obese persons (BMI > 30 kg/m^2^) [229]. LDL cholesterol levels were lowered in 30 healthy people who consumed red beetroot juice (250 mL) together with glucose (300 g). The beetroot juice also lowered the postprandial levels of glucose and lipids [230]. Body weight, body mass index (BMI) and LDL-cholesterol were decreased in obese human volunteers, which consumed freeze-dried red beet leaves (28 g) for 4 weeks [231]. Another study showed that food supplements rich in betalains lowered the total cholesterol, triglyceride, and low-density lipoprotein (LDL) levels in 48 male patients [232]. It was suggested on this basis that betalains may be useful in the management of hyperlipidemia. 

*Hepatoprotective effects*. Animal experiments showed that betanin is effective in the treatment of steatohepatitis, upregulating the peroxisome proliferator-activated receptor (PPAR)-α, downregulating the sterol regulatory element-binding protein (SREBP)-1c, and modifying adipokine levels and lipid profile [233]. Betalain-rich red beet juice decreased hepatic toxicity caused by *N*-nitrosodiethylamine and carbon tetrachloride in the rat [234,235]. Extracts of *Opuntia ficus indica* Mill. fruit protected the liver from damage by carbon tetrachloride and stimulated its recovery in rat experiments [236]. Similar effects reported for betalain-rich extracts of whole plants of *Amaranthus spinosus* were ascribed to the antioxidant activity of the extracts [237]. Induction of detoxification phase II enzyme quinone reductase, improvement of liver redox status, and of mitochondrial functions have been also suggested to contribute to hepatoprotective effects of betalain-rich red beet extract [238] and betanin in particular [239,240]. 

*Neuroprotective effects.* Studies concerning the effects of red beetroots and beetroot extracts on cellular and animal models of neurodegenerative diseases, such as Alzheimer’s disease and Parkinson’s disease (PD) are relatively scarce [241]. Action of 6-hydroxydopamine (6-OHDA) on neuron-like cells is a common cellular model of PD. Betanin (1–200 µM) significantly decreased the 6-OHDA and H_2_O_2_ cytotoxicity and attenuated the ROS level. Betanin (20 and 50 µM) pretreatment protected against 6-OHDA-evoked apoptosis [242]. Betanin has also been reported to attenuate oxidative stress caused by 6-OHDA in PC12 cells and exert antiapoptotic effect via the SAPK/JNK and PI3K pathways. In the rat model of Parkinson’s disease induced by combined administration of tacrine, haloperidol and reserpine, red beet (100, 200 and 300 mg/kg) protected against behavioral changes and ameliorated oxidative stress [243]. These results indicate that betanin may have the ability to prevent or delay the progress of neural death in PD [244].

Betacyanins extracted from *Portulaca oleracea* L. (50 or 100 mg/kg body weight for 2 weeks) reversed the learning and memory impairments induced by d-galactose in mice. Orally administered betalains were more effective than ascorbic acid in reducing cognition deficits in mice, restoring the normal levels of antioxidant enzymes (superoxide dismutases, catalase, glutathione peroxidase and glutathione reductase), and decreasing the level of lipid peroxidation products [245]. 

Betalain-rich aqueous extracts of Swiss chard *Beta vulgaris* L. *var. cicla* inhibited the activity on acetylcholinesterase [246]. This enzyme is important for neurotransmission processes since it decomposes the neurotransmitter acetylcholine and thus limits its action; acetylcholinesterase inhibition has a therapeutic potential in the treatment of neurological disorders, especially Alzheimer’s disease.

Betanin protected against trimethyltin-induced neurodegeneration in mice, including improvement of spatial learning and memory deficits, showing also anxiolytic properties. It protected against neuronal death and neurotransmitter alteration in vulnerable brain areas, including the CA1 region of hippocampus, concurrently with mitigation of behavioral deficits [247]. Excessive consumption of diclofenac and paracetamol is known to induce neurotoxicity and endocrine disruption. In a rat model, supplementation with betanin (25 mg/kg body weight) reduced most of the histopathological and biochemical changes provoked by high doses of these drugs [248]. 

*Cardiovascular effects.* Red beetroot applied as a juice supplement was found to decrease systolic and diastolic blood pressure [36,249,250,251]. For example, 21-day administration of 70 mL of concentrated beetroot juice daily decreased systolic blood pressure after 3 weeks by 7.3 mm Hg on the average; however, the effect disappeared when the supplementation was ceased. A clinical trial confirmed a trend towards lower systolic blood pressure in volunteers who drank daily 500 mL of red beetroot juice [249]. Another study demonstrated lowering of blood pressure in 68 hypertensive persons after drinking 250 mL of beetroot juice daily for 2 weeks [252]. The hypotensive effect can be at least partly ascribed to inhibition of angiotensin I converting enzyme, demonstrated for red beet betalains [41]. In rats fed 3% betalain-rich beetroot crisps (betacyanins, 4.1 mg/g of dry matter; betaxanthins, 2.8 mg/g of dry matter) significant reduction of body weight, blood serum glucose level, cecal atherosclerosis index, isopentric acid concentration were noted [224]. Betalain-rich supplements (50 mg of betalain/betacyanins for 2-weeks) lowered systolic and diastolic blood pressure in human volunteers. Red beetroot betalains significantly lowered blood lipid level without any toxicity and side effects [232].

Sirtuin-1 affects atherosclerosis and probably can prolong the lifespan of human cell so it has been suggested that activation of this protein may prevent cardiovascular diseases. A clinical trial showed that betalains-/betacyanins-rich supplements (50 mg per day for 14 days) increased the levels of sirtuin-1 and decreased the levels of lipoxygenase-1 and C-reactive protein in patients with cardiovascular disease [232]. It seems therefore that betalains can be considered as a food ingredient important for the prevention and treatment of several age-related diseases. 

*Effects in diabetes.* The betalain-rich edible pulps of the prickly pear of different cacti (mainly *Opuntia robusta* or *Opuntia streptacantha* Lem. have been used for generations as a dietary nutrient against diabetes mellitus by Pima Indians in the South of the United States [253]. Indeed, concentrates of beetroot juice with high content of neobetanin, a degradation product of betanin, decreased the postprandial glucose response and insulin secretion, suggesting reduced requirement for insulin [254,255]. A solid betalain formulation prepared from red beet normalized blood glucose level, inhibited transcription of the nuclear factor (NF)-κB and increased sirtuin level; these effects may counteract numerous metabolic dysfunctions [228]. One possible mechanism for the blood glucose lowering effect of red beetroot juice may consist in the inhibition of glucose absorption in the intestine while the decrease of insulin and C-peptide levels induced by the juice can be linked to an increase in cortisol concentration [256]. 

The hypoglycemic effects as well as amelioration of diabetic complications by betalain-rich extracts and betalains have been confirmed in animal experiments. Supplementation of mice fed for 24 weeks an atherogenic diet with betanidin (9.6 mg) for the last 40 days reduced their blood glucose level by 50% [257]. In a diabetic rat model induced by feeding a high-fructose diet, betanin significantly decreased the levels of collagen cross-links and advanced glycation end products (AGEs) and downregulated the receptors for AGEs (RAGEs). In addition, improvement of the redox balance including elevation of levels of antioxidants and antioxidant enzymes was observed. Betanin (25 and 100 mg/(kg*day) decreased markers of glycemia in rats given 30% fructose and ameliorated diabetic cardiac fibrosis [258]. Betanin (25, 50 and 100 mg/(kg*day) ameliorated kidney injury in rat experimental diabetes. This compound can effectively suppress renal fibrosis in diabetic nephropathy and may slow down the progression to end-stage renal disease by regulating TGF-β signaling pathway [259]. Treatment of diabetic rats with aqueous/methanolic extract of the leaves of *B. vulgaris* (50, 100, or 200 mg/kg body weight; 28 days) was reported to reduce the serum glucose level, improve lipid profile, decrease the blood plasma alanine aminotransferase, aspartate aminotransferase, TNF-α, IL-1β, IL-6, hepatic malondialdehyde levels, and increase hepatic triacetyloleandomycin and glutathione levels [260]. Administration of chard (*Beta vulgaris* L. *var. cicla*) extract (2 g/kg body weight for 28 days) increased the number of insulin producing cells in the pancreas of diabetic rats [261]. 

*Anti-inflammatory effects.* Extracts of *Beta vulgaris* dose-dependently suppressed the degradation of tryptophan and the production of neopterin, a marker of activation of the immune system, in human peripheral blood mononuclear cells. The cellular response to stimulation was attenuated by the presence of extracts, demonstrating that the presence of compounds with anti-inflammatory and immunosuppressive activities in the extracts [262]. Lipoxygenase (LOX) and cycloxygenase (COX) are bifunctional enzymes converting arachidonic acid to leukotrienes and prostaglandins, which are mediators of inflammation. Betanidin and betanin were found to inhibit soybean LOX with IC_50_ values of 0.25 and 0.5 μM, respectively, lower than that of catechin (1.1 μM). Betanin (180 μM) inhibited COX-1 and 2 up to 33% and 97%, respectively [218]. Comparison of a range of betalain pigments demonstrated that phenethylamine–betaxanthin is the most potent inhibitor of COX, while betanidin (IC_50_ of 41.4 μM) is an efficient inhibitor of LOX. Docking studies revealed that betalains interact with Tyr-385 and Ser-530 residues close to the active site of COX, and with substrate-binding amino acids of LOX [263]. 

Indicaxanthin and betanin were reported to repress intercellular cell adhesion molecule-1 (ICAM-1), expressed in response to the cytokine treatment increasing intracellular oxidant level in cultured endothelial cells. Betanin was more efficient than indicaxanthin [209]. Another study demonstrated an anti-inflammatory action of gomphrenin I (CAS 17008-59-2) in murine macrophage cell cultures consisting in the suppression of induced production of nitric oxide and decrease in the levels of interleukin (IL)-2β and prostaglandin E2 (PGE2) [192]. Similarly, anti-inflammatory effects of indicaxanthin were found in Caco-2 cell cultures [264] and in vivo in rats with carrageenin-induced pleurisy [193]. The mechanism of the antiinflammatory action of betalains probably involves a reaction cascade resulting in the production of anti-inflammatory cyclopentenone 15-deoxy-PGJ2 [265].

Neutrophils from obese individuals produce more ROS than those from non-obese controls. Beetroot juice and chips attenuated neutrophil oxidative metabolism, decreasing ROS production in a concentration-dependent manner [234]. Betanin (100–500 µM) inhibited lipopolysaccharide-induced activation of microglial cells in vitro, decreasing the production of ROS, reactive nitrogen species, tumor necrosis factor-alpha (TNF-α), interleukin-1 beta (IL-1β) and interleukin-6 (IL-6) [266]. Pro-apoptotic effects of beetroot (0.1–10%) on stimulated neutrophils were also observed in vitro [234]. 

Oral administration of beetroot juice (150 and 300 mg/kg for 28 days) attenuated isoproterenol-induced cardiac dysfunction and structural damage by reducing oxidative stress, inflammation, and apoptosis in the heart. These results provide evidence-based support for the use of beetroot juice in traditional medicine against cardiovascular diseases. Betanin induced the transcription of antioxidant genes through Nrf2 and, simultaneously, suppressed the pro-inflammatory NFĸ-B pathways, thus alleviating endothelial damage and atherogenesis [267]. In a rat model, betanin (25 and 100 mg/(kg*day)) attenuated paraquat-induced lung injury, apparently via antioxidant and anti-inflammatory mechanisms [267]. Betalain (100 mg/kg) reduced also carrageenan-induced recruitment of leukocytes, vascular permeability in the peritoneal cavity, superoxide anion generation by leukocytes, levels of tumor necrosis factor-alpha (TNF-α) and interleukin (IL)-1β in the peritoneal fluid and elevated the level of IL-10. These results suggest that betalain can be considered in the treatment of inflammation-associated diseases [268,269,270]. 

Osteoarthritis (OA) subjects usually suffer progressive discomfort due to pain, stiffness of joints and general tiredness. These conditions are commonly treated with non-steroidal anti-inflammatory drugs (NSAIDS) but efficacy of these drugs is not always satisfactory. In osteoarthritis patients, red beet-concentrate attenuated knee discomfort and joint malfunction [271]. Treatment of OA patients with red beet extract (35–100 mg, twice daily for 10 days) reduced the pain and lowered the serum levels of TNF-alpha and Advanced Oxidation Protein Products (AOPP), suggesting a potential helpfulness of the extract in the management of OA [272]. A patented betalain formulation from red beet extract (betalain content of 24.6%) relieved the feeling of pain in volunteers suffering osteoarthritis by 33%. It has been suggested that this formulation can be also beneficial in such conditions as contact dermatitis, sinusitis, acne and allergy [273].

*Other effects. Opuntia ficus indica* extracts and pure indicaxanthin reduced the contractility of the ileal longitudinal muscle isolated from mice. The probable mechanism of the betaxanthin action involves inhibition of phosphodiesterases causing an elevation of the cAMP level and decrease in intracellular Ca^2+^ concentration, which promotes the smooth muscle relaxation. A potential use of indicaxanthin for the regulation of intestinal motility has been proposed on this basis [274,275].

Juice or extracts of the prickly pear (*Opuntia ficus indica*) attenuated the ulcerogenic effect of ethanol on the stomach in the rat [276]. Similar effects were noted for extracts of flowers of *Opuntia ficus indica f. inermis* [277].

A diuretic effect of dehydrated extract of the *Opuntia ficus indica* fruit, rich in betalains, was comparable to the effect of a standard diuretic hydrochlorothiazide [278]. Red beet roots (*Beta vulgaris*) betalains were reported to exert analgesic effects in different animal models of pain, apparently via modulation of oxidative stress and cytokine levels [279]. 

Numerous reports have dealt with the effects of betalain-containing extracts on the physical performance. Interpretation of these data is not simple since the effects might be due not to betalains but to other compounds present in the extracts, especially nitrates and nitrites. However, some studies point to the action of betalains. Betalain-rich concentrate (BRC) obtained from beetroot containing no sugars or nitrates (100 mg/day given 6 days), was found to improve 10 km run trial performance and recovery in male and female triathletes) as compared with control groups given placebo. The supplemented runners ran faster the 10 km distance. Blood plasma creatine kinase, a marker of muscle damage, increased less after the 10 km run and the increase of subjective fatigue was lower in the group administered with BRC [280]. 

*Beta vulgaris* extract (20–80 mg/kg body weight, 30 days before irradiation and 3 days after irradiation) was reported to have a dose-dependent radioprotective effect in mice exposed to γ radiation. The extract ameliorated radiation-induced changes in several biochemical parameters including activities of main antioxidant enzymes and the level of lipid peroxidation products in the liver, kidneys and spleen, as well as the thymus index and the spleen index. The radioprotective effect was ascribed to the antioxidant activity of the betalains and modulation of the immune system by these compounds [281]. Therapeutic applications of betalains have been summarized in a recent review [244].

Four betalains, (indicaxanthin, indoline carboxylic acid-betacyanin, phenylalanine-betaxanthin, and dopaxanthin) increased the lifespan of *Caenorhabditis elegans* although the effect was rather modest (13–19%). This life extension was due to mitigation of oxidative stress and activation of the transcription factors SKN-1 and DAF-16 (analogs of Nrf2 and FOXO, respectively) [84].

Betalains may participate in maintaining the color of the body in some fish species. Feeding male *Colisa lalia* Hamilton (flame-red dwarf gourami fish) with betalains did not affect the color intensity but allowed for persistence of the color after the periods of social interaction, which was not the case in fish administered with carotenoids and anthocyanins [282].

*Beeturia*. The appearance of red urine and red coloration of feces has been observed in some persons after red beet consumption. This intriguing phenomenon, referred to as beeturia, has been initially linked to iron deficiency since it occurred frequently in iron-deficient subjects and was suspected to be conditioned genetically [283,284,285]. The test used originally to detect beeturia consisted in consumption of 100 g red beets in the evening, emptying the bladder and analysis of urine excreted in the morning. Studies of the population of Glasgow revealed a 13.8% incidence of beeturia, which was associated with low iron level and, consequently, low hemoglobin level [283] although this conclusion has been questioned. Another study associated beeturia with enhanced iron absorption rather than with low iron level [284].

Finally, however, it was established that beeturia is not caused by a genetic trait or disturbed iron level as a single factor but rather due to limited metabolic ability to process betalains from a given food matrix, being a resultant of the amount of the betalain-containing food consumed, simultaneous ingestion of some organic acids such as oxalic acid or ascorbic acid, and the rate of gastric emptying. Thus, beeturia is not a physiological dysfunction but rather a food idiosyncrasy [286,287].

## 8. Betalains as Food Colorants

Betanin from red beet is a commonly used red food colorant. Betanin (Beetroot Red; EEC No. E 162), is the only compound approved for use by the European Union and in the United States under Section 73.40 in the Title 21 of the Code of Federal Regulations (CFR) by the Food and Drug Administration (FDA) as natural red colorant in food products, pharmaceuticals and cosmetics. It has found many applications in the food industry to stain food products and beverages pink, red or violet [34,153,288,289,290,291,292] (Table S6). As a rule, less than 50 mg betanin/kg is sufficient to produce the desired color, but some food experts have suggested the necessity of use of higher quantities of betanin (0.1–1.0% *w/w*) [293]. Red beet-based colorants are commercially available as either juice concentrates (obtained by vacuum concentration of red beet juice to 60–65% total solids) or powders, of pigment content of 0.3–1% [294,295]. Betalains are well soluble in water, which makes them very convenient as food supplements [50]. Red beet betalains constituted 10% of global food colorants in 2009 [296].

Foods containing fat, especially meats, are endangered by the lipid peroxidation process, which adversely affects their sensorial properties, nutritional value and shelf life, and produces compounds potentially harmful to health. In order to inhibit this process, addition of synthetic antioxidants, especially BHA and BHT, is employed in the food industry [297,298]. However, these antioxidants may exert deleterious health effects themselves, since they have been suspected to be tumor promoters on the basis of animal experiments [299,300]. Therefore, a trend toward application of natural antioxidants instead of synthetic ones is being observed [301]. Betalains are compounds of natural origin having the advantage of being colorants and antioxidants with a long record of beneficial health effects so there are no basic counterindications for their application in the food industry.

However, red beet products have some disadvantages: the contain mainly betalain so the range of colors they can produce is narrow. Moreover, their earthy-like flavor due to the presence of geosmin and some pyrazines is unpleasant and may be not desirable especially in dairy products [40,302]. Other betalain sources have thus been explored. From among betalain-containing plants, the most promising are cacti (the family *Cactaceae*), especially prickly pears (genus *Opuntia*) and pitayas (genera *Cereus, Hylocereus* and *Selenicereus*). They have minimal water and soil requirements, can be cultivated in arid and semi-arid regions [153] and their fruits are good sources of betalains [303,304], Unlike the red beetroot, cactus fruits do not have unpleasant flavor and their betalains represent a broader spectrum of colors, from yellow–orange (*Opuntia* sp.) to red–violet (*Hylocereus* sp.). As yellow water-soluble pigments are scarce, the fruits of *Opuntia* containing both betacyanins (0.001–0.059%) and betaxanthins (0.003–0.055%) [190] are especially valuable [8,14,77,80,190,305,306,307]. Fruits of various *Opuntia* cacti have different betacyanin/betaxanthin ratios [174,306], and fruits not containing betacyanins are not known [306]. In turn, *Hylocereus* fruits have high concentrations (0.23–0.39%) of nonacylated and acylated betacyanins, and, unlike beetroots, are devoid of betaxanthins [69,118]. 

Due to the relatively low stability and risk of decomposition, and consequently change of color, betalains cannot be used in products subject to the action of light, oxidants and metal ions, heated at high temperatures, and in highly acid products. Therefore, they are used most frequently as an additive in yoghurts, jellies and cold drinks [34,64]. 

## 9. Food Safety

Most of the studies concerning biological activities, health effects, bioavailability and dietary safety of betalains have been performed on red red beet betalains [255,308,309]. These studies demonstrated that betalains are generally safe; as a result, the use of red beet betanin as food colorant has been approved. One study reported that intravenous injection of red beet betanin provoked a transient increase of blood pressure and heart rate [195]. However, such effects have not been observed when betalains were administered per os, perhaps due to degradation in the GI tract [197]. Betanin did not stimulate and even inhibited production of IgE and IgG, which evidences the lack of allergic response to the pigment [310]. 

Red beet pigments did not initiate or promote hepatocarcinogenesis [311]. They showed no genotoxicity or mutagenicity in the Ames test or short-term toxicity so no contraindications for their applications exist [312,313]. All studies performed to date indicated the safety of betalains for human consumption, although data regarding embryotoxicity, teratogenicity and generational toxicity, are still lacking. 

Safety of a variety of new sources of betalains has been also tested. Betalain-rich extracts from fruits of some *Cactaceae* species did not show mutagenicity in the Ames test [314]. Betalain-rich extracts from *Hylocereus polyrhizus* [315], *Myrtillocactus geometrizans* fruits [197] and *Rivina humilis* berry [18] did not cause any toxic effects in rodents and are unlikely to be toxic to humans. However, some potential sources are known to be unsafe as the contain compounds having undesired activities. For example, *Phytolacca decandra* L. berries contain toxic saponins, and for this reason the berries are not utilized commercially [316]. Significant amounts of dopamine (41 μmol/g fresh weight) are present in *Celosia argentea* var. *cristata*, which may cause problems upon ingestion of extracts of this plant [80].

## 10. Other Applications

Reversible changes in betalain fluorescence upon metal binding may enable detection of metals, among them Cu^2+^ and Eu^2+^ and of compounds which chelate metals such as dipicolinic acid and, therefore, of endospores of health-threatening species, including *Bacillus anthracis* [317].

Addition of *Hylocereus polyrhizus* extract to into starch/polyvinyl alcohol films used for shrimp package improved the antioxidant and antimicrobial abilities of the films, increased the sensitivity of the film to ammonia and enabled monitoring of the freshness of shrimp (“intelligent packaging”) [318]. Betalain-containing films show good UV barrier properties [319]. Betalains have been proposed for an eco-friendly way of dyeing of fibrous materials such as textiles or leather [320].

Due to their light absorbing properties and to their capacity to transfer electrons, betalain-containing extracts or pure betalains have been used in dye-sensitized solar cells [321]. I.a., betalain-rich extracts from fruit and root of pokeweed (*Phytolacca americana* L.), *Beta vulgaris* and *Bougainvillea* sp. have been used [322,323]. Dye-sensitized solar cells containing various natural pigments have been proposed. Cells employing anthocyanins and carotenoids have solar energy conversion efficiencies lower than 1%, while betalain containing cells can reach conversion efficiencies of up to 1.7%, which is comparable to that of plant photosynthesis [324,325,326]. Optimization of dye-sensitized solar cells using purified betanin instead of raw extracts allowed for increasing the energy conversion efficiencies up to 2.7% [327,328]. 

## 11. Environmental Role and Fate of Betalains 

Betalains provide color mainly flowers and fruits so they may play a role as attractants for pollinators (insects or birds) and for seed dispersal [35]. The role of betalains in other parts of plants (e.g., leaves, stem, root) is not obvious but undoubtedly betalains may protect against pathogens. Tobacco plants bioengineered to produce betalain producing were significantly more resistant toward gray mold (*Botrytis cinerea*), a pathogen responsible for major losses in tobacco plantations [53]. Hydrogen peroxide [329], light and UV radiation [330], drought, salinity and low temperature [327,331,332,333,334] stimulate betalain synthesis, which suggests that betalains have a role in defense against these agents as antioxidants, light and UV absorbing agents and contributors to osmotic equilibrium playing a role similar to proline [335]. An increase in the betalain content was also reported in response to wounding [336]. Betalains were reported to be produced in injured tissues, normally not pigmented, perhaps as a defense mechanism against infection [337]. Generally, betalain-producing plants are more suitable for survival in harsh environments than related species not containing betalains [335]. It has been also suggested that can deter herbivores [338]. There are no data on the role in betalains in coloration of animals although they might contribute to color maintenance as demonstrated in fish [282].

Betalains, produced by the plants and mushrooms, are consumed and digested by herbivores. Part of them is absorbed and metabolized, part is excreted in feces, and a small fraction in urine. In rats, betalains of the garambullo are almost completely degraded in the gastrointestinal tract within 24 h but absorbed betalains are eliminated via urine without being metabolized in the liver [186,195,197]. Betalains are also released from dying plants. The released betalains seem to be rapidly decomposed by plant enzymes or soil microbes as no accumulation of these pigments in the soil has been reported.

Red beetroot extracts show an allelopathic action, inhibiting the growth of *Phalaris minor* and *Malva parviflora* [339]; however, it is an open question if betalains may participate in such interactions. The mechanism of betalain degradation in the environment and their possible role in allelopathic interactions await elucidation.

## 12. Concluding Remarks

Betalains occur in many plants, they are relatively easy to obtain in more-or-less pure forms, are more stable than anthocyans and anthocyanidins at near-neutral pH and are good candidates to replace, whenever possible, synthetic colorants. The use of the latter is often a compromise between esthetic effects and possible dangers for health while betalain may bring health-promoting effects in addition to color. In addition, they find applications outside the food industry. However, their health-promoting effects need further studies on pure compounds since reports on the effects of fruits or juices may be biased by the contribution of other, non-betalain components present in these materials. Betalains find also ingenious applications outside the food industry.

## Figures and Tables

**Table 1:** Factors affecting betalain stability.

Factors Enhancing Stability	Factors Decreasing Stabilty
Presence of matrix	Absence of matrix
High pigment concentration	Low pigment concentration
Low water activity	High water activity
High extent of glycosylation	Low extent of glycosylation
High extent of acyltion	Low extent of acylation
3 < pH < 7	pH < 3 or pH > 7
Low temperature	High temperature
Darkness	UV and light
Absence of oxygen	Oxygen
Antioxidants	H_2_O_2_, other oxidants
Metal chelators	Metal cations
	Degrading enzymes

## Supplementary Materials

The following are available online. Tables S1–S6. Table S1: Structures and Absorption Maxima of Betacyanins; Table S2: Structures and Absorption Maxima of Betaxanthins; Table S3: Quantum Yield Φ of Fluorescence of Betalains; Table S4: Fluorescence Properties of Some Betalain Pigments; Table S5: The Content of Betalains and Other Antioxidants in Various *Opuntia* ssp. Clones; Table S6: Some Food Applications of Betalains from Red Beetroot.
