# Supplementary material for: Biological Properties and Applications of Betalains
Source: Molecules. 2021 Apr 26;26(9):2520. doi: 10.3390/molecules26092520 (PMC8123435; doi:10.3390/molecules26092520)
Supplement: Supplementary file 1 [file molecules-26-02520-s001.zip › molecules-1149306-Supplementary.pdf]

Table S1.

Structures and Absorption Maxima of Betacyanins. Based on [49], modified.

Compound	Source	Structure	Absorption maximum [nm]	Reference(s)
Betanidin (1)<br>CAS 2181-76-2	*Beta vulgaris* L.	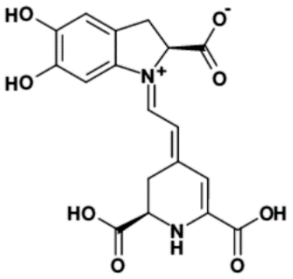	541	[340]
Betanin (2)<br>CAS 7659-95-2	*Beta vulgaris* L.; *Ullucus tuberosus* Caldas	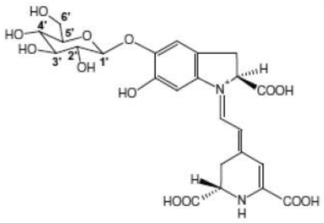	537;<br>535	[341]<br>[66, 342]
2′-*O*-Apiosyl-betanin (3)<br>2′-*O*[5″-*O*-(E)-feruloyl]-apiosyl-betanin (4)<br>2′-*O*[5″-*O*-(E)-sinapoyl]-apiosyl-betanin (5)	*Hylocereus ocamponis* (Salm-Dyck) Britton & Rose<br>*Phytolacca americana* L.<br>*Hylocereus ocamponis* (Salm-Dyck) Britton & Rose	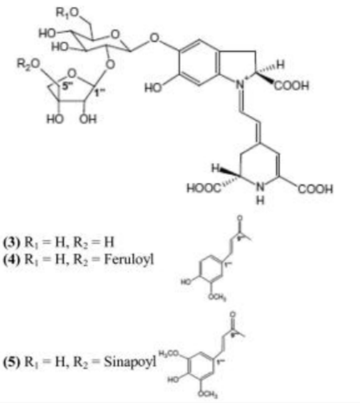	539<br>331, 548<br>330,550	[343]<br>[30, 344]<br>[30]
Phyllocactin (6)<br>Betanidin-5-O-(6′-O-malonyl)-β-glucoside<br>CAS 15167-85-8	*Phyllocactus hybridus* Hort.; *Ullucus tuberosus* Caldas	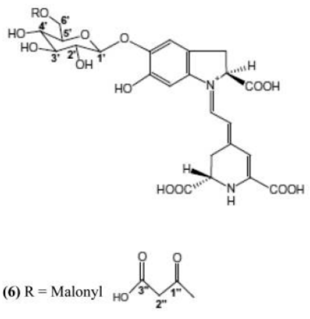	539<br>535	[160]<br>[342]

2′-*O*-apiosyl-phyllocactin (7)<br><br>2′-*O*-[5″-*O*-(*E*)-feruloyl]-apiosyl-phyllocactin (8)	Christmas cactus<br>*Schlumbergera x buckleyi*	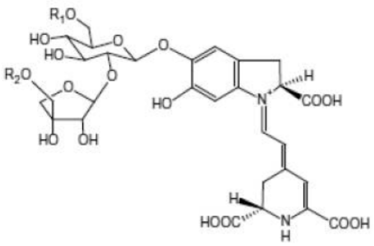<br>**(7)** R_1 = Malonyl, R_2 = H<br>**(8)** R_1 = Malonyl, R_2 = Feruloyl	538<br><br>328, 549	[345]<br><br>[344]
4′-*O*-Malonyl-betanin (9)	*Hylocereus ocamponis* Britton & Rose	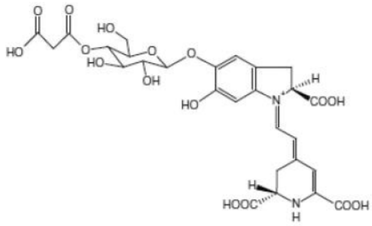	538	[343]
2-Descarboxybetanidin (10)	*Carpobrotus acinacoformis* (L.) L.Bolus	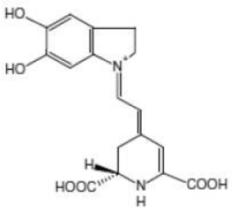	533	[66]
2-Descarboxybetanin (11)<br><br>6′-O-Malonyl-2-descarboxybetanin (12)	*Beta vulgaris* L.<br><br>*Beta vulgaris* L.	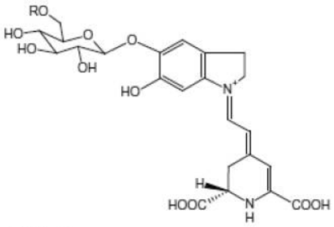<br>**(11)** R = H<br>**(12)** R = Malonyl	532<br><br>535	[66]<br><br>[66]
Hylocerenin (13)<br>CAS 403517-96-4	*Hylocereus polyrhisus* (F.A.C. Weber) Britton & Rose	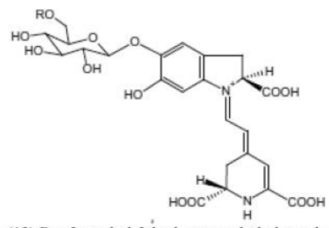<br>**(13)** R = 3-methyl-3-hydroxy methyl glutaryl =<br>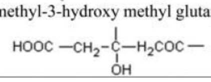	541	[69]

Lampranithin I (14)<br>Lampranithin II (15)	*Lampranthus sp.*<br>*Lampranthus sp.*	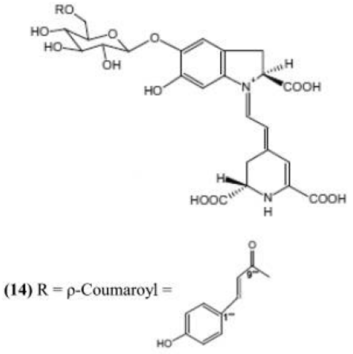<br>(14) R = ρ-Coumaroyl =<br>(15) R = Feruloyl	290, 320, 538<br>288, 322, 538	[346]<br>[346, 347]
Prebetanin (16)<br>CAS 13798-16-8<br>Rivinianin (17)<br>CAS 58115-21-2	*Beta vulgaris* L.<br>*Phytolacca americana* L.<br>*Rivina humilis* L.	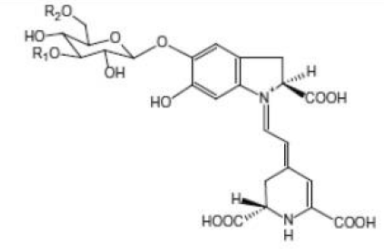<br>(16) R_1 = H, R_2 = HSO_3<br>(17) R_1 = HSO_3, R_2 = H	538<br>235, 541	[344]<br>[348, 349]
Neobetanin (18)<br>CAS 71199-29-6	*Beta vulgaris* L.	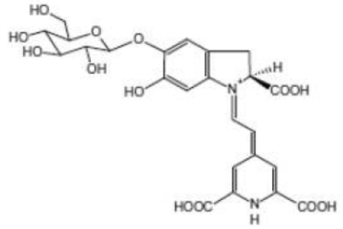<br>(18) R_1 = glucosyl, R_2 = H	267, 306, 470	[67]
Gomphrenin I (19)<br>CAS 17008-59-2<br>Gomphrenin II (20)<br>CAS 143022-02-0<br>Gomphrenin III (21)<br>Gomphrenin IV (22)	*Gomphrena globosa* L.<br>*Gomphrena globosa* L.<br>*Gomphrena globosa* L.<br>*Ullucus tuberosus* Caldas<br>*Gomphrena globosa* L.	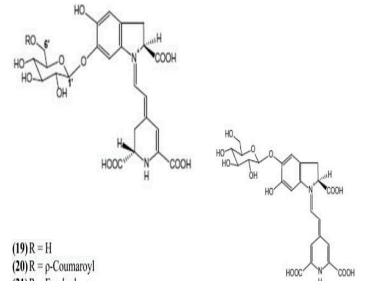<br>(19) R = H<br>(20) R = p-Coumaroyl<br>(21) R = Feruloyl<br>(22) R = Sinapoyl<br>(18) R_1 = glucosyl, R_2 = H	543<br>550;<br>547<br>550	[350]<br>[237, 342]<br>[351]<br>[16]

Amaranthin (23)<br>CAS 15167-84-7<br>Iresinin I (25)<br>CAS 78413-55-5<br>Celosianin I (25)<br><br>Celosianin II (26)<br>CAS 114847-18-6<br>Sinapoyl-amaranthin (27)	*Celosia cristata* L.<br><br>*Iresine herbstii* Hook.<br>*Celosia argentea* L. var. *cristata*<br>*Celosia argentea* L. var. *cristata*<br>*Gomphrena globate* L.	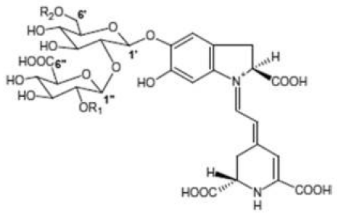<br>(23) R_1 = H, R_2 = H<br>(24) R_1 = H, R_2 = 3-hydroxy-3-methyl glutaryl = $H_3C-CH_2-CH(OH)-H_2COC-$<br>(25) R_1 = H, R_2 = ρ-Coumaroyl<br>(26) R_1 = H, R_2 = Feruloyl<br>(27) R_1 = H, R_2 = Sinapoyl	536<br>298, 540<br>306, 546<br>312, 546<br>540	[65, 80]<br>[352]<br>[352]<br>[352]<br>[16]
Bougainvillein-r I (28)<br>CAS 30513-63-4<br>Bougainvillein-r III (28)<br>Feruloyl- bougainvillein-r I (28)	*Bougainvillea ssp.*<br><br>*Bougainvillea ssp.*<br><br>*Ullucus tuberosus* Caldas	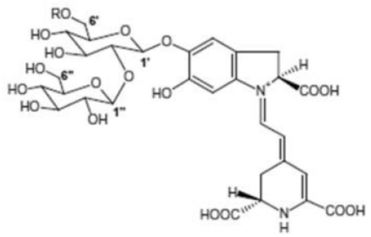<br>(28) R = H<br>(29) R = ρ-Coumaroyl<br>(30) R = Feruloyl	538<br>312, 541<br>534	[13, 250]<br>[353]<br>[17]
Mammillarinin (31)<br>4′-*O*-(E)-Malonyl-bougainvillein-r I (32)	*Mammillaria ssp.*<br>*Mammillaria ssp.*	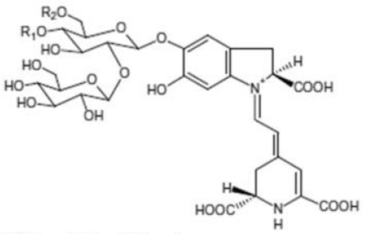<br>(31) R_1 = H, R_2 = Malonyl<br>(32) R_1 = Malonyl, R_2 = H	539<br>538	[349]<br>[349]
2-Descarboxy-mammillarinin (33)	*Mammillaria ssp.*	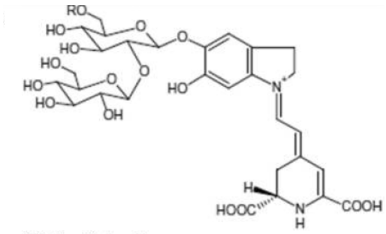<br>(33) R = Malonyl	533	[349]

			540	[354]
	Bougainvillea glabra Choisy<br>*Bougainvillea glabra* Choisy		316, 545	[112]
Bougainvillein-v (34)<br>6′-*O*-(E)-Caffeoyl-Bougainvillein-v (35)<br>6′-*O*-(E)-p-Coumaroyl-Bougainvillein-v (36)<br>6′′-*O*-(E)-p-Coumaroyl-Bougainvillein-v (37)<br>6′,6′′-di--*O*-(E)-p-Coumaroyl-Bougainvillein-v (38)<br>6′′-O-Ramnosyl-Bougainvillein-v (39)	*Bougainvillea glabra* Choisy	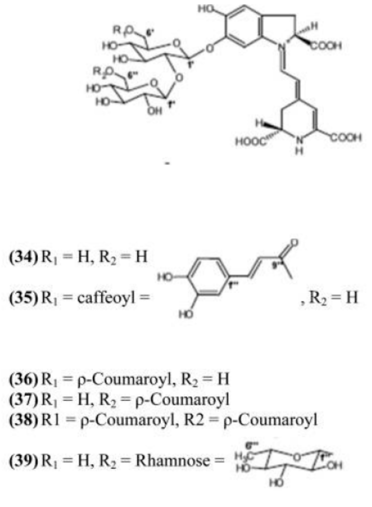	306, 540	[112]
	Bougainvillea glabra Choisy		306, 540	[112]
	Bougainvillea glabra Choisy		307, 548	[112]
	Bougainvillea glabra Choisy var. *sanderiana*		541	[355]
2″-*O*{[6′-*O*-(E)-Caffeoyl]-[6″-*O*-(E)-*p*-coumaroyl]}-glucosyl- bougainvillein-v (40)<br>2″-*O*[6,6′′-di-*O*-(E)-coumaroyl]-glucosyl-bougainvillein-v (41)<br>2″-*O*{[6′-*O*-(E)-Caffeoyl]-[6″-*O*-(E)-*p*-coumaroyl]}-sophorosyl-bougainvillein-v (42)	*Bougainvillea glabra* Choisy	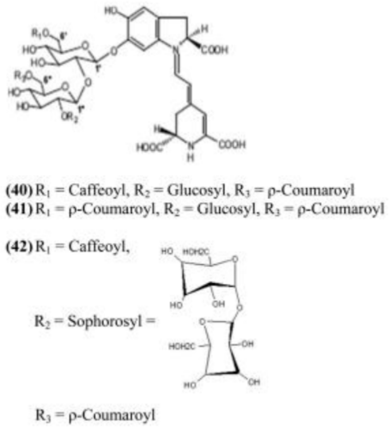	312, 547	[112]
	Bougainvillea glabra Choisy		307, 548	[112]
	Bougainvillea glabra Choisy		312, 549	[112]

Isobetanin-5-O-β-glucoside	*Ullucus tuberosus* Caldas	535	[342]
Dehydro-phyllocactin	*Ullucus tuberosus* Caldas	538	[342]
Dehydro-isophyllocactin	*Ullucus tuberosus* Caldas	537	[342]
Betanidin-5-O-(4′-O-malonyl-β-glucoside)	*Ullucus tuberosus* Caldas	535	[342]
Isobetanidin-5-O-(6′-O-malonyl)-β-glucoside (isophyllocactin)	*Ullucus tuberosus* Caldas	535	[342]
Isobetanidin-5-O-(4′-O-malonyl)-β-glucoside	*Ullucus tuberosus* Caldas	535	[342]
2-Decarboxy-phyllocactin	*Ullucus tuberosus* Caldas	533	[342]
Betanidin-feruloyl-5-O-β-diglucoside	*Ullucus tuberosus* Caldas	533	[342]
Isobetanidin-feruloyl-5-O-β-diglucoside	*Ullucus tuberosus* Caldas	532	[342]
Betanidin-5-O-(6′-O-feruloyl)-β-glucoside (lampranthin II)	*Ullucus tuberosus* Caldas	540	[342]
Isobetanin-5-O-(6′-O-feruloyl)-β-glucoside (isolampranthin II)	*Ullucus tuberosus* Caldas	540	[342]
Isobetanin-6-O-(6′-O-feruloyl)-β-glucoside (isogomphrenin III)	*Ullucus tuberosus* Caldas	547	[342]

Additional References:

340. Wyler, H.; Mabry, T.J.; Dreiding, A.S. Zur Struktur des Betanidins-Uber die konstitution des Randenfarbstoffes Betanin. *Helv. Chim. Acta* **1963**, *46*, 1745–1748.
341. Wyler, H.; Dreiding, A.S. Kristallisiertes Betanin. Vorlaufige Mitteilung. *Helv. Chim. Acta* **1957**, *40*, 191–192.
342. Mosquera, N.; Cejudo-Bastante, M.J.; Heredia, F.J.; Hurtado, N. Identification of New Betalains in Separated Beta-cyanin and Betaxanthin Fractions from Ulluco (*Ullucus tuberosus* Caldas) by HPLC-DAD-ESI-MS. *Plant Foods Hum. Nutr*. **2020**, *75*, 434–440.
343. Wybraniec, S.; Nowak-Wydra, B. Mammillarinin: A new malonylated betacyanin from fruits of *Mammillaria*. *J. Agric. Food Chem*. **2007**, *55*, 8138–8143.
344. Schliemann, W.; Joy, I.V.; Komamine, R.W.; Metzger, A.; Nimtz, M.; Wray, V.; Strack, D. Betacyanins from plants and cell cultures of *Phytolacca americana*. *Phytochemistry* **1996**, *42*, 1039–1046.
345. Kobayashi, N.; Schmidt, J.; Nimtz, M.; Wray, V.; Schliemann, W. Betalains from Christmas cactus. *Phytochemistry* **2000**, *54*, 419–426.
346. Piattelli, M.; Impellizzeri, G. Betacyanins from *Lampranthus* sp. (*Aizoaceae*). *Phytochemistry* **1969**, *8*, 595–1596.
347. Strack, D.; Bokern, M.; Marxen, N.; Wray, V. Feruloylbetanin from petals of *Lampranthus* and feruloylamaranthin from cell suspension cultures of *Chenopodium rubrum. Phytochemistry* **1988**, *27*, 3529–3531.
348. Kujala, T.; Loponen, J.; Pihlaja, K. Betalains and phenolics in red beetroot (*Beta vulgaris*) peel extracts: Extraction and characterization. *Z. Naturforsch.* **2001**, *56*, 343–348.
349. Imperato, F. Betanin 3′-sulphate from *Rivinia humilis*. *Phytochemistry* **1975**, *14*, 2526–2527.
350. Heuer, S.; Strack, D. Synthesis of betanin from betanidin and UDP-glucose by a protein preparation from cell sus-pension cultures of *Dorotheanthus bellidiformis* (Burm. f.) NE. Br. *Planta* **1992**, *186*, 626–628.
351. Gläßgen, W.E.; Metzger, J.W.; Heuer, S.; Strack, D. Betacyanins from fruits of *Basella rubra. Phytochemistry* **1993**, *33*, 1525–1527.

352. Cai, Y.; Sun, M.; Corke, H. Identification and distribution of simple and acylated betacyanins in the *Amaranthaceae*. *J. Agric. Food Chem.* **2001**, *49*, 1971–1978.
353. Piattelli, M.; Imperato, F. Betacyanins from *Bougainvillea. Phytochemistry* **1970**, *9*, 455–458.
354. Piattelli, M.; Imperato, F. Pigments of *Bougainvillea glabra. Phytochemistry* **1970**, *9*, 2557–2560.
355. Imperato, F. A branched trisaccharide in the betacyanins of *Bougainvillea glabra. Phytochemistry* **1975**, *14*, 2526.

Table S2. Structures and Absorption Maxima of Betaxanthins. From [49], modified

General formula:

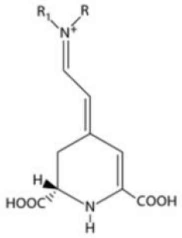

Pigment	Source	R	R1	Absorption maximum [nm]	References
Indicaxanthin<br>CAS 2181-75-1	*Opuntia ficus-indica* L.		Proline	260, 305, 485	[9, 356]
Portulacaxanthin I<br>CAS 11042-69-6	*Portulaca grandiflora* Hook.<br>*Opuntia ficus-indica* L.		Hydroxyproline	483	[62, 357]
Portulacaxanthin II<br>CAS 135545-98-1	*Portulaca grandiflora* Hook.<br>*Beta vulgaris* L.	H	Tyrosine	468	[14, 24]
Portulacaxanthin III	*Portulaca grandiflora* Hook.<br>*Beta vulgaris* L.; *Ullucus tuberosus* Caldas	H	Glycine	470<br><br>469	[14, 24]<br><br>[342]
Vulgaxanthin I<br>CAS 904-62-1	*Beta vulgaris* L.; *Ullucus tuberosus* Caldas	H	Glutamine	470<br>467	[9, 358]<br>[342]
Vulgaxanthin II<br>CAS 1047-87-6	*Beta vulgaris* L.	H	Glutamic acid	469	[14, 358]
Vulgaxanthin III	*Beta vulgaris* L. var. *lutea*<br>*Beta vulgaris* L. ssp. *cicla*	H	Asparagine	470<br>457	[14]<br>[322, 359]
Vulgaxanthin IV	*Beta vulgaris* L. var. *lutea*<br>*Beta vulgaris* L. ssp. *cicla*	H	Leucine	470	[14, 359]
Miraxanthin I<br>CAS 5296-79-7	*Mirabilis jalapa* L.	H	Methionine sulfoxide	475	[4, 360]

Miraxanthin II<br>CAS 5375-63-3	*Mirabilis jalapa* L.	H	Aspartic acid	477	[4, 360]
Miraxanthin III<br>CAS 5589-85-5	*Mirabilis jalapa* L.	H	Tyramine	473.5	[4, 14]
3-Methoxytyramine-Bx	*Mirabilis jalapa* L.<br>*Celosia argentea* L. *var. cristata*	H	3-Methoxytyramine	461	[4, 80]
Miraxanthin V<br>CAS 5375-64-4	*Mirabilis jalapa* L.<br>*Beta vulgaris* L.<br>*Celosia argentea* L. *var. cristata*	H	Dopamine	475.5	[4, 66]
Histamine-Bx	*Mirabilis jalapa* L.<br>*Beta vulgaris* L.	H	Histamine	468	[4, 14]
Dopaxanthin<br>CAS 71199-31-0	*Glotiphyllum longum* (Haw.) N.E.Br.	H	L-DOPA	472	[63]
Humilixanthin<br>CAS 111534-70-4	*Rivina humilis* L.	H	Hydroxynorvaline	258, 463, 483	[361]
α-Aminobutyric acid-Bx	*Beta vulgaris* L.	H	α-Aminobutyric acid	459	[9]
Methylated arginine-Bx	*Amaranthus tricolor* L.	H	Methyl derivative of arginine	478	[362]
(*S*)-Tryptophan-Bx	*Celosia argentea var. cristata*	H	(*S*)-Tryptophan	218, 264, 471	[196]
Serine-Bx	*Beta vulgaris* L.	H	Serine	468	[9]
Valine-Bx	*Beta vulgaris* L.	H	Valine	470	[9]
Phenylalanine-Bx	*Beta vulgaris* L.	H	Phenylalanine	472	[9]
Isoleucine-Bx	*Beta vulgaris* L.	H	Isoleucine	470	[9]
Alanine-Bx	*Beta vulgaris* L. *ssp. cicla* [L.]	H	Alanine	468	[14]
Musca-aurin I<br>CAS 52012-51-8	*Amanita muscaria* (L.) Lam.)	H	Ibotenic acid		[27, 28]
Muscimol-Bx	*Amanita muscaria* (L.) Lam.)	H	Muscimol	214, 264, 469	[28]
4,5-Dihydromuscimol-Bx	*Amanita muscaria* (L.) Lam.)	H	4,5-Dihydromuscimol	260, 466	[28]
Musca-aurin II<br>CAS 12624-17-8	*Amanita muscaria* (L.) Lam.)	H	Stizolobic acid	196, 260, 473	[27, 28]
Musca-aurin VII<br>CAS 81943-08-0	*Amanita muscaria* (L.) Lam.)<br>*Beta vulgaris* L. *ssp. cicla* [L.]	H	Histidine	472	[14, 27]
Methionine-Bx	*Opuntia sp.*	H	Methionine	477	[364]
Threonine-Bx	*Beta vulgaris* L. *ssp. cicla* [L.]; *Ullucus tuberosus* Caldas	H	Threonine	469<br>465	[324, 364]

Arginine-Bx	*Gomphrena globose* L.; *Ullucus tuberosus* Caldas	H	Arginine	469<br>470	[16]<br>[342]
Lysine-Bx	*Gomphrena globose* L.	H	Lysine	458	[17]
Ethanolamine-Bx	*Beta vulgaris* L. *ssp. cicla* [L.]	H	Ethanolamine	460	[364]
Putrescine-Bx	*Bougainvillea ssp.*	H	Putrescine	461	[16]
Phenylethylamine-Bx	*Opuntia ssp.*	H	Phenylethylamine	475	[357]

Additional References:

356. Impellizzeri, G.; Piattelli, M. Biosynthesis of indicaxanthin in *Opuntia ficus-indica* fruits. *Phytochemistry* **1972**, *11*, 2499–2502.
357. Castellanos-Santiago, E.; Yahia, E.M. Identification and quantification of betalains from the fruits of 10 Mexican prickly pear cultivars by high performance liquid chromatography and electrospray ionization mass spectrometry. *J. Agric. Food Chem*. **2008**, *56*, 5758–5764.
358. Piattelli, M.; Minale, L.; Nicolaus, R.A. Ulteriori ricerche sulle betaxantine. *Rend. Accad. Sci. Fis. Mat. Naples* **1965**, *32*, 55–56.
359. Hempel, J.; Böhm, H. Betaxanthin pattern of hairy roots from *Beta vulgaris* var. *lutea* and its alteration by feeding of amino acids. *Phytochemistry* **1997**, *44*, 847–852.
360. Gandía-Herrero, F.; García-Carmona, F.; Escribano, J. Development of a protocol for the semi-synthesis and purification of betaxanthins. *Phytochem. Anal*. **2006**, *17*, 262–269.
361. Strack, D.; Schmitt, D.; Reznik, H.; Boland, W.; Grotjahn, L.; Wray, V. Humilixanthin a new betaxanthin from *Rivina humilis. Phytochemistry* **1987**, *26*, 2285–2287.
362. Biswas, M.; Dey, S.; Sen, R. Betalains from *Amaranthus tricolor* L. *J. Pharmacogn. Phytochem*. **2012**, *1*, 88–96.
363. Cai, Y.Z.; Sun, M.; Corke, H. Characterization and application of betalain pigments from plants of the *Amaranthaceae. Trends Food Sci. Technol*. **2005**, *16*, 370–376.
364. Kugler, F.; Graneis, S.; Stintzing, F.C.; Carle, R. Studies on betaxanthin profiles of vegetables and fruits from the *Chenopodiaceae* and *Cactaceae. Z. Naturforsch. C J. Biosci*. **2007**, *62*, 311–318.

Table S3. Quantum Yield Φ of Fluorescence of Betalains

**Betalain**	**CAS number**	**Φ in water**	**Φ in methanol**	**Φ in ethylene glycol**	**Reference**
Betanin	7659-95-2	0.0007	0.0013	0.0047	[79]
Indicaxanthin	2181-75-1	0.0053	0.0081	0.033	[79]
Vulgaxanthin I	904-62-1	0.0073	0.011	0.039	[365]
Miraxanthin V	5375-64-4	0.003	0.0047	0.015	[366]
Miraxanthin I	5296-79-7	0.0084	-	-	[80]

Additional References:

365. Niziński, S.; Wendel, M.; Rode, M.F.; Prukała, D.; Sikorski, M.; Wybraniec, S.; Burdziński, G. Photophysical properties of betaxanthins: Miraxanthin V—Insight into the excited-state deactivation mechanism from experiment and computations. *RSC Adv*. **2017**, *7*, 6411–6421.
366. Wendel, M.; Szot, D.; Starzak, K.; Tuwalska, D.; Gapinski, J.; Naskrecki, R.; Prukala, D.; Sikorski, M.; Wybraniec, S.; Burdzinski, G. Photophysical properties of betaxanthins: Vulgaxanthin I in aqueous and alcoholic solutions. *J. Luminesc*. **2015**, *167*, 289–295.

Table S4. Fluorescence Properties of Some Betalain Pigments. From [47], modified.

Betalain	CAS number	Amino acid	λ_{ex} (nm)	λ_{em} (nm)	Stokes shift (nm)	Reference
Betanin	7659-95-2		535	608	73	[328]
Indicaxanthin	2181-75-1	Pro	463	515	52	[6, 74]
Vulgaxanthin I	904-62-1	Gln	464	509	45	[6, 74]
Vulgaxanthin II	1047-87-6	Glu	466	508	42	[74]
Dopaxanthin	71199-31-0	DOPA	463	510	47	[6, 74]
Miraxanthin I	5296-79-7	MetSO	475	509	34	[74]
Miraxanthin II	5375-63-3	Asp	474	507	33	[74]
Miraxanthin III	5589-85-5	tyramine	464	506	42	[74]
Miraxanthin V	5375-64-4	dopamine	465	512	47	[6, 74]
Musca-aurin VII	81943-08-0	His	465	509	44	[74]
Portulacaxanthin II	135545-98-1	Tyr	474	509	35	[6, 74]
Alanine-Bx		Ala	463	508	45	[74]
Methionine-Bx		Met	464	509	45	[74]
Vulgaxanthin IV		Leu	464	509	45	[74]
Phenylalanine-Bx		Phe	464	510	46	[74]
Phenelethylamine-Bx		phenelethylamine	473	551	78	[47]
		ethylamine	472	548	76	[47]
		propylamine	472	548	76	[47]
		N-methylethanamine	472	548	76	[47]
		N-methyl-N-propylamine	472	550	78	[47]
		pyrrolidine	471	549	78	[47]
		aniline	513	560	47	[47]
		N-methylaniline	494	553	59	[47]
		N-ethylaniline	494	554	60	[47]
		indoline	521	570	49	[47]
		(*S*)-indoline-2-carboxylic acid	529	575	46	[47]

Table S5. The Content of Betalains and Other Antioxidants in Various *Opuntia* ssp. Clones. According to [190], modified.

Cultivar	Total phenolics as gallic acid equivalents [mg/L]	Betaxanthins as indicaxanthin equivalents [mg/L]	Betacyanins as betalain equivalents [mg/L]	Ascorbic acid [mg/L]	TEAC		ORAC	
					Juice [mmol/L]	Pulp [mmol/kg]	Juice [mmol/L]	Pulp [mmol/kg]
Green	243 ± 13.4	0.4 ± 0.02	0.1±0.01	51.1±3.0	3.31±0.13	2.24±0.09	5.45	3.68
Orange	247 ± 23.1	76.3 ± 0.38	6.6±0.04	70.2±16.0	3.10±0.04	2.32±0.03	5.83	4.36
Red	335 ± 19.3	67.9 ± 0.19	120.0±0.44	67.9±16.5	3.71±0.47	2.60±0.33	6.35	4.44
Purple	660 ± 35.8	195.8 ± 0.46	431.0±1.04	95.4±0.6	4.99±0.37	3.64±0.27	11.20	8.16

Table S6. Some Food Applications of Betalains from Red Beetroot

Food product	Betalain additive	Results	Addition level (w/w)	Reference
Yoghurt	Beetroot powder into the yogurt	Optimal sensory acceptance, rheological, and physicochemical properties	2%	[367]
Ice cream	Beetroot juice	Decreased apparent viscosity, dry matter and overrun, but high biofunctional properties	2.5%, 5%, and 10%	[368]
Baked goods	Beetroot powder	Good hydration properties and viscometric properties of wheat dough, physical characteristics and sensory attributes of baked rolls	2–10%	[369]
Biscuits	Beetroot extract	Increased nutritional values, acceptability and bioactivities.	7	[370]
Jellies	Beetroot juice	Good quality jelly	Beetroot juice was heated with 2% pectin, 61% sugar, and 0.5% citric acid	[371]
Candies	Beetroot pomace	Improved phytochemical properties of the candies	9.24%	[372]
Chicken frankfurters	Beetroot juice concentrate	Good color stability without adversely affecting flavor or texture	0.48%	[373]
Pasta	Beetroot	Addition of beetroot can improve the quality of pasta, and render attractive color on the pasta	150 g/kg	[374]
Noodles	Beetroot pulp	Addition of beetroot improves the color, nutritional, and sensory properties of formulated noodles, and evokes an increment in antioxidant activity.	10–40% Noodles	[375]
Meat	Red beetroot powder	Increased antioxidant activity and extended shelf life	0.15%	[376]
Nondairy drinks	Fresh beetroot	Beetroot drink was a good product without cholesterol and contained health promoting components		[377]
Fish	Red beet peel extracts	Extension of the shelf life of rainbow trout, improved chemical and sensory quality of fish	0.1% (w/v) ice with red beet peel extracts	[378]
Sausages	Betalain powder from beetroot	The color of betalain-containing sausages proved to be more stable toward light exposure during storage than the color of those containing nitrite-nitrate salts	33, 45, 56 ppm	[379]

Beverages	Beetroot	Decreased nephrotoxicity	4 g/L	[380]

Additional References:

367. Dabija, A.; Codina, G.G.; Ropciuc, S.; Stroe, S.G. Studies regarding the production of a novel yogurt using some local plant raw materials. *J. Food Process. Preserv*. **2019**, *43*, e13826.
368. Ozturk, G.; Dogan, M.; Said Toker, O. Physicochemical, functional and sensory properties of mellorine enriched with different vegetable juices and TOPSIS approach to determine optimum juice concentration. *Food Biosci*. **2014**, *7*, 45–55.
369. Kohajdova, Z.; Karovicova, J.; Kuchtova, V.; Laukova, M. Utillisation of beetroot powder for bakery applications. *Chem. Pap*. **2018**, *72*, 1507–1515.
370. Amnah, M.A. Nutritional, sensory and biological study of biscuits fortified with red beetroots. *Life Sci. J.* **2013**, *10*, 1579–1584.
371. Chaudhari, S.N.; Nikam, M.P. Development and sensory analysis of beetroot jelly. *Int. J. Sci. Res*. **2015**, *4*, 827–830.
372. Kumar, V.; Kushwaha, R.; Goyal, A.; Tanwar, B.; Kaur, J. Process optimization for the preparation of antioxidant rich ginger candy using beetroot pomace extract. *Food Chem*. **2018**, *245*, 168–177.
373. Vareltzis, K.P.; Buck, E.M. Color stability and sensory attributes of chicken frankfurthers made with betalains and potassium sorbate versus sodium nitrite. *J. Food Prot*. **1984**, *47*, 41–45.
374. Rekha, M.N.; Chauhan, A.S.; Prabhasankar, P.; Ramteke, R.S.; Rao, G.V. Influence of vegetable purees on quality attributes of pastas made from bread wheat (*T. aestivum). CyTA J. Food* **2013**, *11*, 142–149.
375. Chhikara, N.; Kushwaha, K.; Sharma, P.; Gat, Y.; Panghal, A. Bioactive compounds of beetroot and utilization in food processing industry: A critical review. *Food Chem*. **2019**, *272*, 192–200.
376. Xiao, C.G.; Tan, L.L.; Zhu, P.P.; Yang, H.J.; Chen, D.; Lu, W.J.; Ren, F.Z.; Guo, H.Y.; Ge, S.Y.; Tang, H.G.; et al. The coloring effect and antioxidant effect of red beet in minced pork. *J. Chin. Inst. Food Sci. Technol.* **2019**, *19*, 111–121.
377. Panghal, A.; Virkar, K.; Kumar, V.B.; Dhull, S.; Gat, Y.; Chhikara, N. Development of probiotic beetroot drink. *Curr. Res. Nutr. Food Sci*. **2017**, *5*, 257–262.
378. Yavuzer, E.; Ozogul, F.; Ozogul, Y. Impact of icing with potato, sweet potato, sugar beet, and red beet peel extract on the sensory, chemical, and microbiological changes of rainbow trout (*Oncorhynchus mykiss*) fillets stored at (3 ± 1 °C). *Aquacult. Int*. **2020**, *28*, 187–197.
379. Van Elbe, J.H.; Klement, J.T.; Amundson, C.H.; Cassens, R.G.; Lindsay, R.C. Evaluation of betalain pigments as sausage colorants. *J. Food Sci*. **1974**, *39*, 128–132.
380. Iahtisham-Ul-Haq, Butt, M.S.; Randhawa, M.A.; Shahid, M. Nephroprotective effects of red beetroot-based beverages against gentamicin-induced renal stress. *J. Food Biochem*. **2019**, *43*, 12873.
